# Supplementary material for: A novel generative framework for designing pathogen-targeted antimicrobial peptides with programmable physicochemical properties
Source: PLoS Comput Biol. 2025 Dec 29;21(12):e1013833. doi: 10.1371/journal.pcbi.1013833 (PMC12747415; doi:10.1371/journal.pcbi.1013833)
Supplement: S1 Appendix — (PDF) [file pcbi.1013833.s001.pdf]

S1 Details of Model

S1.1 CVAE

S1.1.1 Encoder Architecture

The encoder receives the amino acid sequence as input. First, each amino acid in the sequence is mapped to a 128-dimensional learnable vector (embedding layer), and a positional encoding is added to let the model know the relative order of each position in the sequence. Next, the sequence representation is passed into a network composed of a 3-layer stacked Transformer encoder. Each layer contains multi-head self-attention (4 attention heads) and a feed-forward network (128→512→128), and includes standard residual connections and LayerNorm. This is consistent with the classical Transformer encoder structure, except that the scale is deliberately compressed to a smaller configuration (3 layers, 128 dimensions, 4 heads) in order to make subsequent generation more computationally efficient.

This encoder does not simply encode the sequence alone, but rather performs encoding under conditional attributes. The model additionally receives an attribute vector (dimension 10), and then broadcasts this property vector along the temporal dimension so that every position in the sequence "sees" the same property description. After that, this property vector is concatenated with the 128-dimensional representation at every position, and then passed through a linear layer to project the concatenated representation back down to 128 dimensions. This linear mapping layer is essentially a dedicated fusion layer that injects the global attribute into the per-position representation, so that the Transformer encoder learns an attribute-conditioned sequence representation.

S1.1.2 Latent Space

After Transformer encoding, the model produces a representation with a shape similar to [batch size, sequence length, 128-dimensional features]. Instead of using a standard VAE approach where the entire sequence is flattened and directly compressed into a single vector via fully connected layers, this model uses a bottleneck structure based on 1D convolution plus pooling. Specifically, the model converts the sequence features into a format suitable for convolutional processing, and then applies multiple stages of 1D convolution and max pooling to progressively compress the sequence length

while extracting local fragment-level motif information. In the end, the downsampled convolutional features are flattened into a fixed-length global vector, which is then mapped by linear layers to form the latent representation. In other words, the latent variable is not a pure global average; it is a compact representation that still encodes local sequence patterns. This is the rationale for replacing simple fully connected compression with convolutional operations: along the sequence dimension, convolution shares weights, making it more parameter-efficient at capturing local structure, and progressive downsampling makes it possible to compress the entire sequence into a compact global descriptor.

S1.1.3 Decoder Architecture

The task of the decoder is to regenerate the peptide sequence from the latent variable. As a first step, the model takes the latent vector and applies a linear transformation followed by a series of transposed convolutions to progressively upsample it, expanding the original global vector back into a pseudo-memory sequence, i.e., stretching the compressed global representation back into a time-resolved sequence of features with approximately the original length. This step can be understood as the inverse of the convolutional bottleneck: the encoder squeezes the sequence into a compact representation, and the decoder unfolds it back into a contextual memory that can be consumed by the generator.

Subsequently, the model again concatenates the same property vector to this upsampled memory sequence and then projects it back to the standard hidden dimensionality through the same linear fusion layer. In other words, the property is not only injected into the representation on the encoder side; it is re-injected on the decoder side as well, and it directly conditions the memory that the decoder uses for cross-attention. This means that when the decoder predicts each subsequent amino acid, it is always attending to a context that already carries the desired attribute information. Therefore, generation is conditional generation by construction, rather than generate first and filter later.

Something very important also happens during decoding: the decoder is not only generating the sequence, it is also attempting to reconstruct or predict the input attribute itself. We define a small fully connected prediction head that reads the hidden states of the decoder and outputs a 10-dimensional attribute vector. During training, this prediction is compared against the true

attribute, and the resulting error is backpropagated through the entire decoder, then further back through the memory sequence (which is obtained by expanding the latent variable), then back into the latent space, and ultimately also influences the encoder that produced the latent representation. This design has two advantages: first, it forces the latent space and the decoder to explicitly preserve attribute information, enabling controllable generation; second, it ensures that the attribute is not treated as an external tag, but instead acts as a conditioning signal that participates in both the forward path and the backward path.

S1.2 Diffusion Model

S1.2.1 Denoising Network Architecture

Our denoising network is a lightweight Bert Encoder tailored specifically for this task. The network consists of three stacked self-attention encoder layers. Each layer contains a multi-head self-attention sub-layer and a position-wise feed-forward sub-layer, together with residual connections and LayerNorm. The overall structure follows the standard Bert Encoder design. Each layer uses a hidden size of 768 and 6 attention heads, meaning that the hidden representation is split into 6 subspaces for attention and then recombined. Compared to a conventional BERT-base model (12 layers, 12 heads), we explicitly reduce the depth to 3 layers and the number of heads to 6 in order to decrease parameter count and computation cost, which makes the model efficient enough to run inside the iterative denoising/sampling loop of the diffusion process.

Although our implementation reuses the transformers.models.bert.modeling_bert. BertEncoder module, it is important to emphasize that we do not load any BERT pretrained weights from natural language tasks, nor do we rely on any large external text corpora. All parameters (including attention weights, feed-forward layers, and LayerNorm parameters) are randomly initialized and trained end-to-end jointly with the diffusion denoising objective. In other words, our model is not a conventional "fine-tuned BERT," but rather a Transformer with BERT-style architecture used as the backbone of the diffusion denoiser. Its representational capacity is learned entirely from our task-specific data distribution.

S1.2.2 Fusion of Diffusion Timestep and Class Conditioning

Our diffusion model is a conditional generative model: it must know not only “which diffusion step we are currently at” (i.e., the noise level), but also “which target class distribution we aim to denoise toward” (for example, positive vs. negative, in a binary setting). Both types of conditioning information are encoded as 768-dimensional global vectors and are injected into the input sequence representation via element-wise addition at every sequence position. In our design, the class-conditioning vector and the timestep-conditioning vector are combined in the same representation space, so that class control directly influences the denoising trajectory itself, rather than being applied as a post hoc filter.
