# Supplementary material for: A novel generative framework for designing pathogen-targeted antimicrobial peptides with programmable physicochemical properties
Source: PLoS Comput Biol. 2025 Dec 29;21(12):e1013833. doi: 10.1371/journal.pcbi.1013833 (PMC12747415; doi:10.1371/journal.pcbi.1013833)
Supplement: S2 Appendix — (PDF) [file pcbi.1013833.s002.pdf]

S2 Basic Analysis of AMPs

To preliminarily validate the rationality of our generated AMPs, we analyzed the length and the amino acid composition of the generated peptide sequences. These key properties can reflect whether the designed AMPs are reasonable.

As shown in Fig S1, the AMPs targeting both pathogens are predominantly concentrated within the 20–25 amino acid range, accounting for over 50% of the total sequences. This suggests that the lengths of the generated peptides fall within an optimal range for antimicrobial activity. Notably, AMPs targeting *S. aureus* exhibit a significantly higher proportion in the 25–30 residue range compared to those targeting *E. coli*, while sequences in the 15–20 and 35–40 ranges are slightly more prevalent among *E. coli*-targeting AMPs. These differences reflect the varying structural or mechanistic requirements of AMPs against different bacterial targets. In Fig S2, we compare the

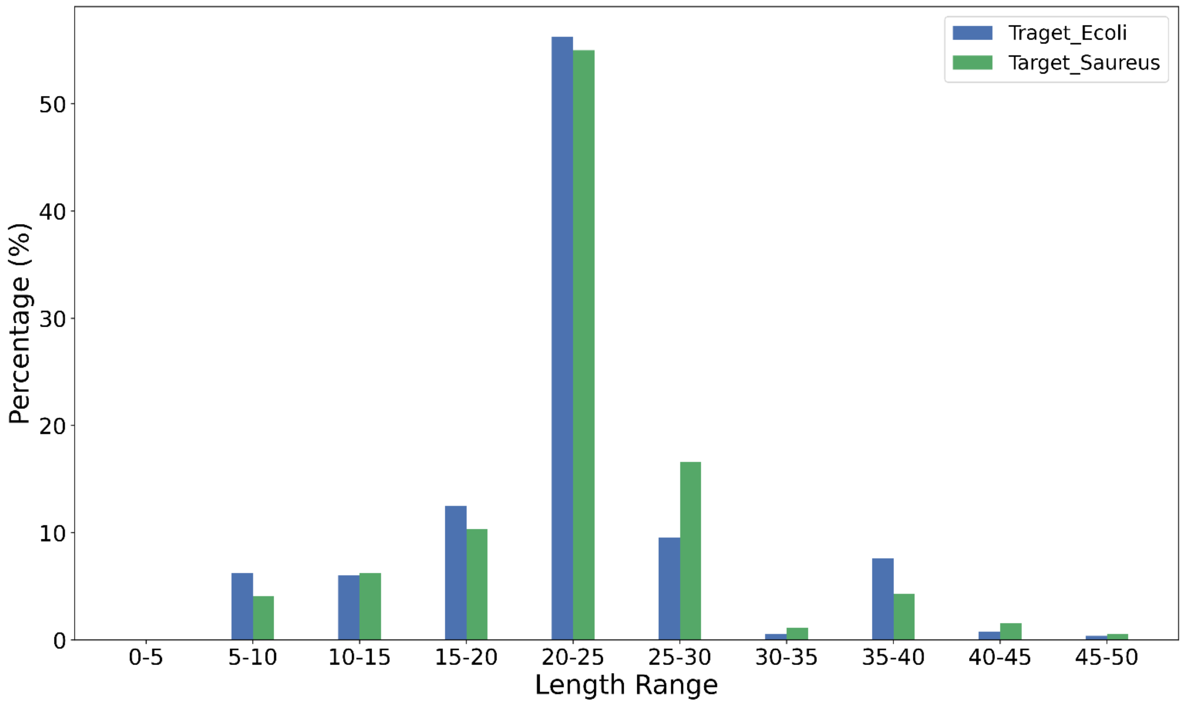

Fig S1: Comparison of distribution of AMPs lengths.

amino acid composition of natural and generated AMPs for both *S. aureus* and *E. coli* targets. Overall, the distributions are broadly consistent across groups. However, the proportions of amino acids such as K (Lysine), C (Cysteine), and A (Alanine) in the generated sequences are lower than those in the natural sequences, while the proportions of E (Glutamic), I (Isoleucine), and M

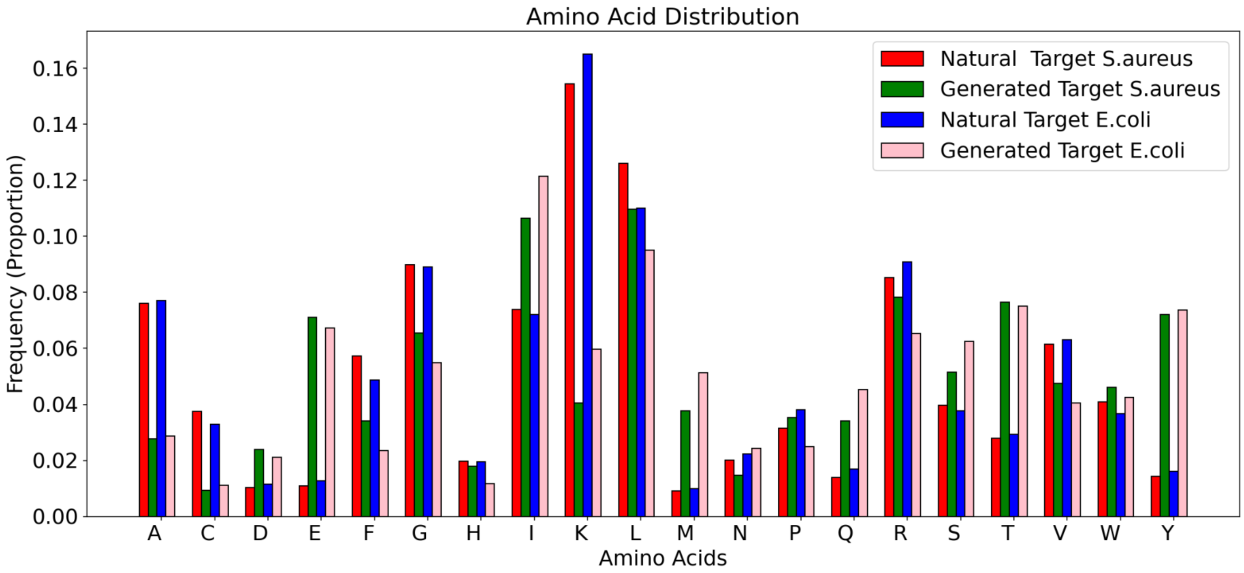

Fig S2: Comparison of amino acid distribution.

(Methionine) are higher. This indicates that the generative model selectively emphasizes features associated with antimicrobial activity. While the overall compositional profiles remain close to those of natural AMPs, these local discrepancies may highlight the model's trade-off strategy between preserving natural sequence characteristics and optimizing functional performance.
