# Supplementary material for: A novel generative framework for designing pathogen-targeted antimicrobial peptides with programmable physicochemical properties
Source: PLoS Comput Biol. 2025 Dec 29;21(12):e1013833. doi: 10.1371/journal.pcbi.1013833 (PMC12747415; doi:10.1371/journal.pcbi.1013833)
Supplement: S3 Appendix — (PDF) [file pcbi.1013833.s003.pdf]

S3 Comparison of Pre-Trained and Directly Trained Models

To evaluate the effectiveness of our method when trained on a larger peptide sequence dataset, we conducted a comparative analysis of property retention performance between a model trained from scratch and one initialized with pretraining on the large dataset.

As shown in Fig S1, the pretrained model exhibited a significantly higher property retention rate of 91.2% (defined by a mean squared error (MSE) threshold of < 0.0625), in contrast to only 68.2% achieved by the non-pretrained model. This corresponds to a notable improvement of 23 percentage points.

These results underscore the critical role of pretraining. By leveraging knowledge acquired from a broader sequence space, the pretrained model is better able to preserve essential biochemical and physicochemical properties during sequence generation. This demonstrates that pretraining enables more stable and property-aware generation, which is particularly important for tasks such as AMP design, where the preservation of functional attributes is crucial.

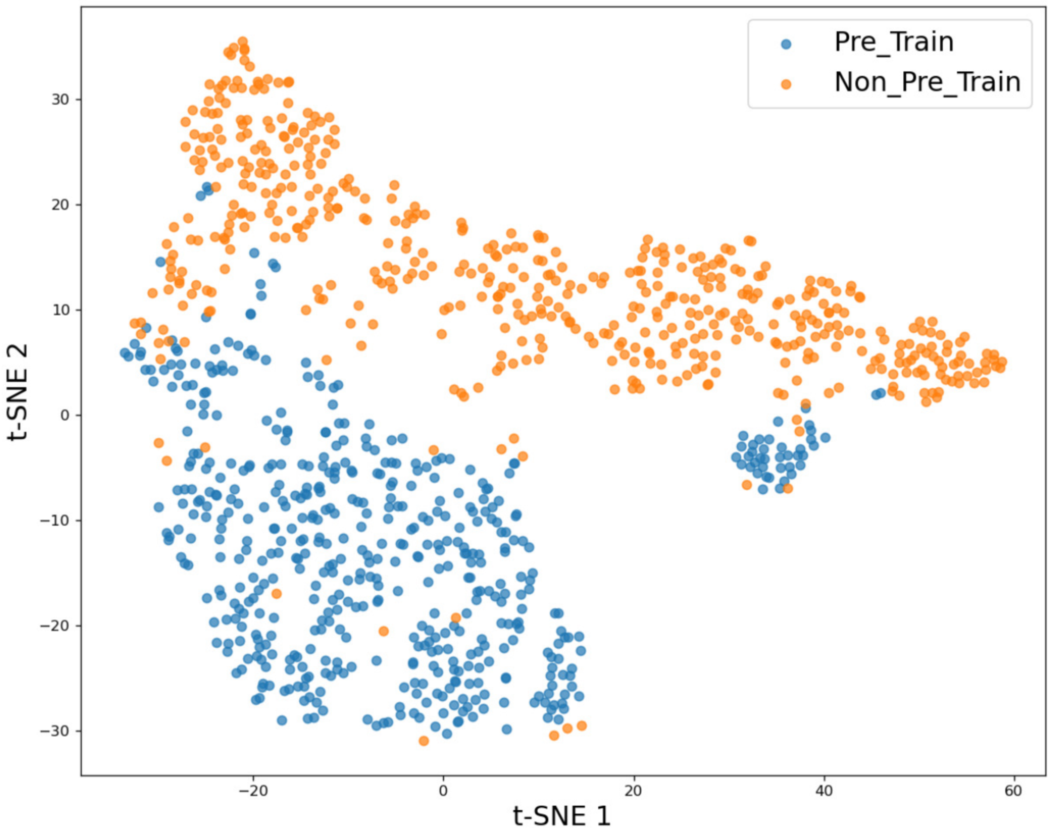

Fig S1: Comparison of Attribute Retention in Pre-Trained and Directly Trained Models.
