# Supplementary material for: A novel generative framework for designing pathogen-targeted antimicrobial peptides with programmable physicochemical properties
Source: PLoS Comput Biol. 2025 Dec 29;21(12):e1013833. doi: 10.1371/journal.pcbi.1013833 (PMC12747415; doi:10.1371/journal.pcbi.1013833)
Supplement: S4 Appendix — (PDF) [file pcbi.1013833.s004.pdf]

S4 Training Details

This section presents the training details of each stage in our multi-phase training process, including the pretraining of the CVAE (Fig S2), the pretraining (Fig S1) and fine-tuning (Fig S3) of the diffusion model, and the training of the MIC predictor (Fig S4).

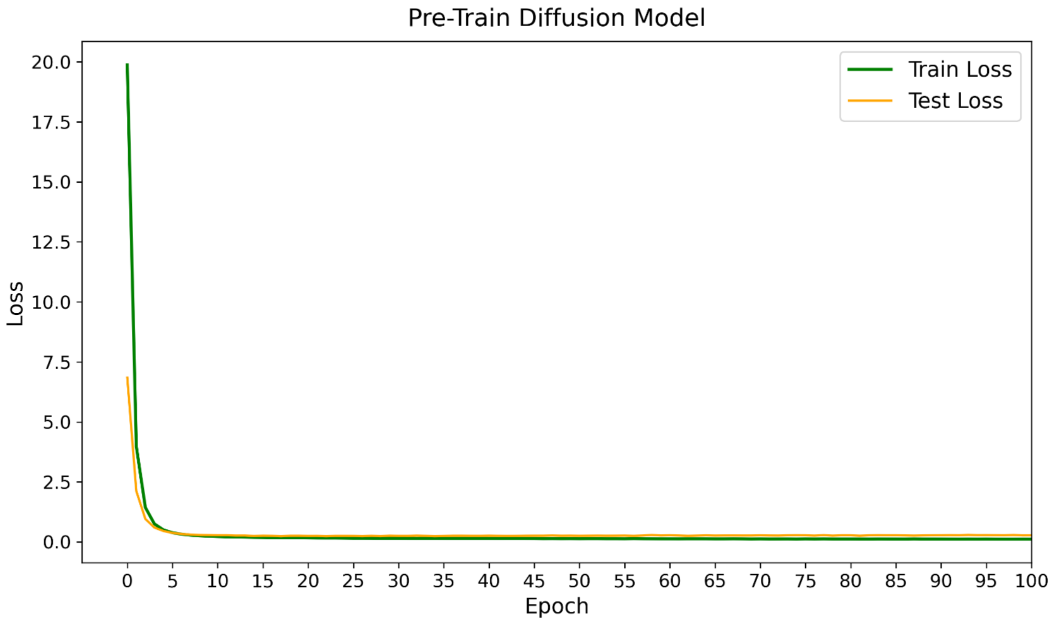

Fig S1: Loss Curve of Pre-trained Diffusion Model.

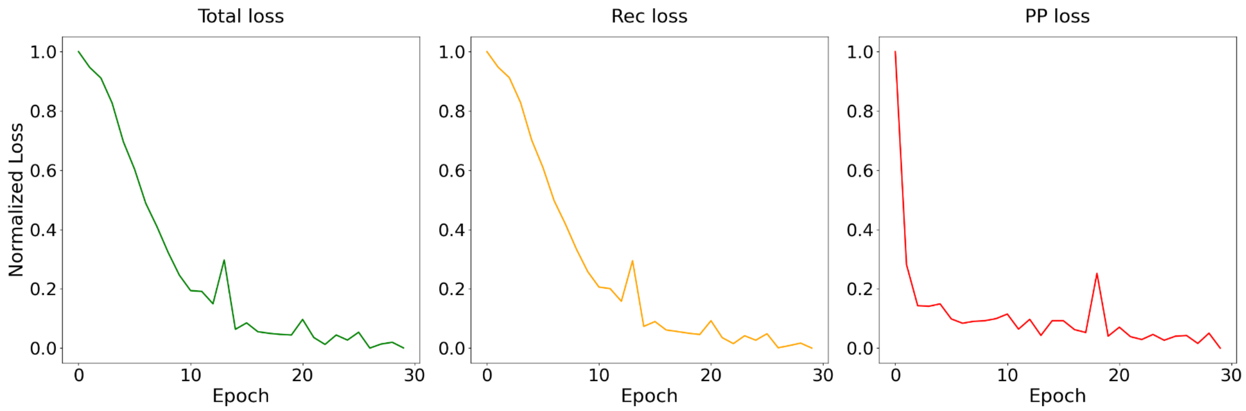

Fig S2: Loss Curve of Pre-trained Conditional Variational Autoencoder. The Total loss represents the overall loss of the CVAE, as defined in Eq. 1 of the main text. Rec Loss refers to the reconstruction loss, while PP Loss corresponds to the property preservation loss.

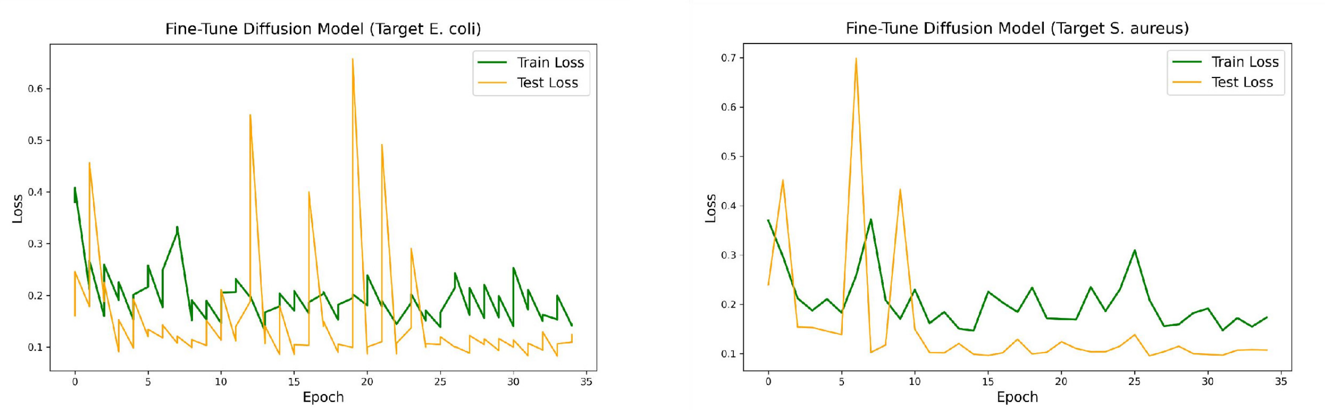

Fig S3: Loss Curve of Fine-Tune Diffusion Model.

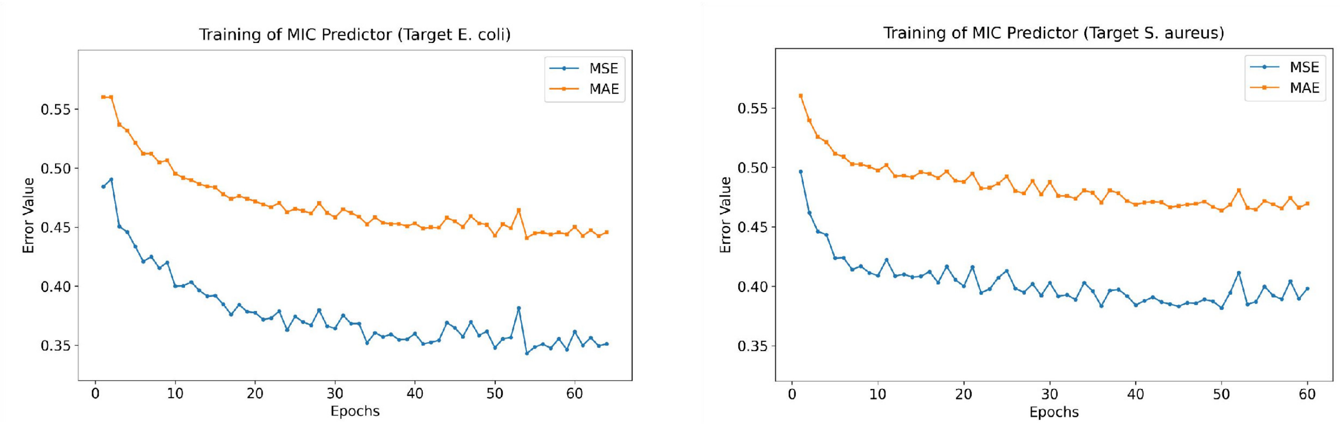

Fig S4: Loss Curve of Trained MIC Predictor.
