# Supplementary material for: A novel generative framework for designing pathogen-targeted antimicrobial peptides with programmable physicochemical properties
Source: PLoS Comput Biol. 2025 Dec 29;21(12):e1013833. doi: 10.1371/journal.pcbi.1013833 (PMC12747415; doi:10.1371/journal.pcbi.1013833)
Supplement: S5 Appendix — (PDF) [file pcbi.1013833.s005.pdf]

S5 Physicochemical Properties Setting

The normalized maximum and minimum data are shown in Table S1. The property settings for targeting two groups of bacteria are shown in Table S2. The AMP attributes with large attribute differences between the two groups are shown in Table S3. The cecropin and improved property values are shown in Table S4.

Table S1: Min and Max Values for Feature Normalization.

	MIN Value	MAX Value
Molecular Weight	75.0666	6908.82
Isoelectric Point (pI)	4.05	12.0
GRAVY	-4.5	4.5
Aromaticity	0.0	1.0
Instability Index	-112.24	485.94
Disulfide Bonds	0.0	66000.0
Molecular Volume	60.1	8185.2
α-Helix	0.0	1.0
β-Sheet	0.0	1.0
Random Coil	0.0	1.0

Table S2: Target Attributes of Generated Antimicrobial Peptides.

	E. coli	*S. aureus*
Molecular Weight	0.36	0.34
Isoelectric Point (pI)	0.83	0.81
GRAVY	0.48	0.50
Aromaticity	0.12	0.13
Instability Index	0.24	0.24
Disulfide Bonds	0.08	0.08
Molecular Volume	0.38	0.36
α-Helix	0.37	0.41
β-Sheet	0.16	0.16
Random Coil	0.20	0.22

Table S3: Property Values of Two Selected AMPs.

	AMP1	AMP2
Molecular Weight	0.25	0.29
Isoelectric Point (pI)	0.79	0.80
GRAVY	**0.63**	**0.29**
Aromaticity	**0.06**	**0.33**
Instability Index	0.17	0.19
Disulfide Bonds	**0.08**	**0.41**
Molecular Volume	0.28	0.30
α-Helix	**0.56**	**0.33**
β-Sheet	0.12	0.13
Random Coil	0.06	0.00

Table S4: Property Values of Cecropin and Cecropin-Improve.

	Cecropin	*Cecropin_Improve*
Molecular Weight	0.47	0.47
Isoelectric Point (pI)	0.82	0.82
GRAVY	0.43	0.43
Aromaticity	**0.03**	**0.23**
Instability Index	0.23	0.23
Disulfide Bonds	0.08	0.08
Molecular Volume	0.49	0.49
α-Helix	**0.22**	**0.42**
β-Sheet	0.29	0.29
Random Coil	0.25	0.25
