# Supplementary material for: A novel generative framework for designing pathogen-targeted antimicrobial peptides with programmable physicochemical properties
Source: PLoS Comput Biol. 2025 Dec 29;21(12):e1013833. doi: 10.1371/journal.pcbi.1013833 (PMC12747415; doi:10.1371/journal.pcbi.1013833)
Supplement: S6 Appendix — (PDF) [file pcbi.1013833.s006.pdf]

# S6 Docking Score

This part removes the docking results of the star AMP from the top 20. As can be seen from Fig S1 and S2, the docking values and confidence scores of these peptide sequences with the cell membrane remain in an optimal range, indicating the effectiveness of our generated peptides.

Specifically, Seq1-1, Seq2-7, and Seq2-8 all exhibited docking scores below –300 and confidence scores exceeding 0.96. Although they showed slightly inferior performance in hemolytic activity, which excluded them from being classified as top-performing AMPs, these sequences still demonstrate promising potential as candidate peptides.

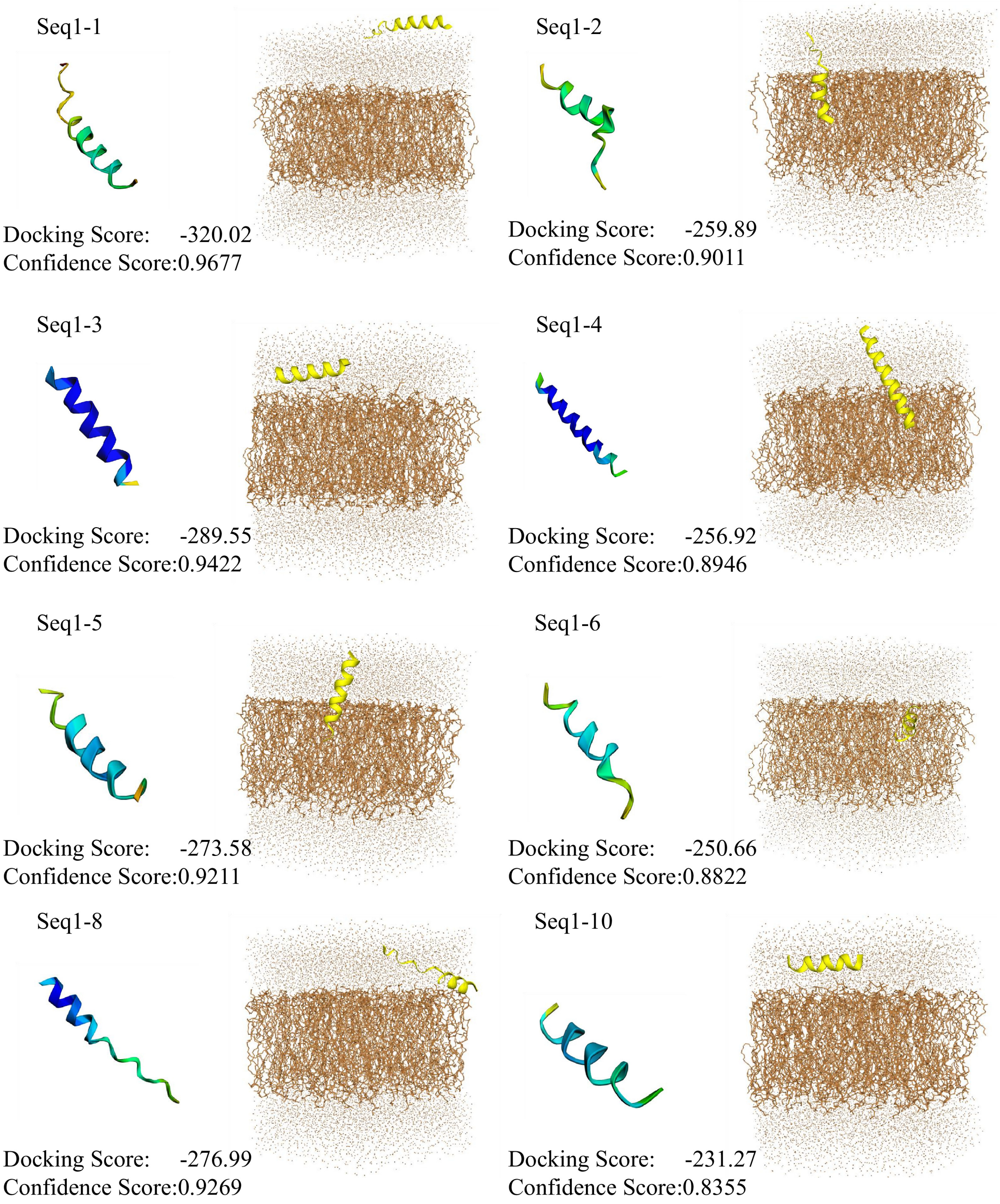


**Fig S1**: Molecular Docking of AMP with *E. coli* Cell Membrane. Docking score (from ITScorePP/ITScorePR): more negative means stronger predicted binding; values around -200 or lower are considered strong. Confidence score: above 0.7 suggests high likelihood of binding, between 0.5 and 0.7 suggests possible binding, and below 0.5 suggests low likelihood.

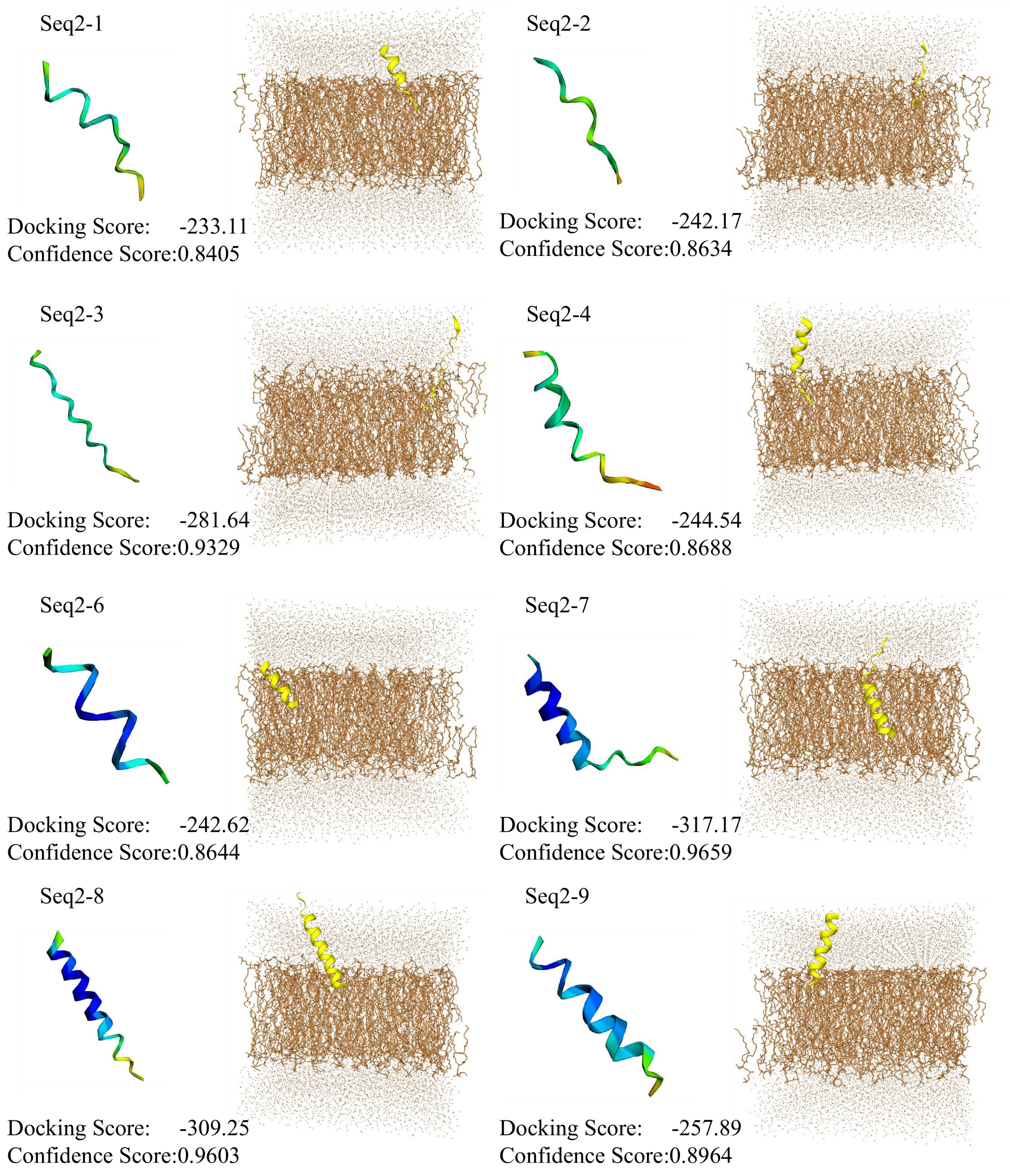


**Fig S2**: Molecular Docking of AMP with *S. aureus* Cell Membrane. Docking score (from ITScorePP/ITScorePR): more negative means stronger predicted binding; values around -200 or lower are considered strong. Confidence score: above 0.7 suggests high likelihood of binding, between 0.5 and 0.7 suggests possible binding, and below 0.5 suggests low likelihood.
