# Supplementary material for: A novel generative framework for designing pathogen-targeted antimicrobial peptides with programmable physicochemical properties
Source: PLoS Comput Biol. 2025 Dec 29;21(12):e1013833. doi: 10.1371/journal.pcbi.1013833 (PMC12747415; doi:10.1371/journal.pcbi.1013833)
Supplement: Attachment — Submitted filename: 202510_PLOSCB.pdf [file pcbi.1013833.s007.pdf]

What are the main claims of the paper and how significant are they for the discipline?

- In this paper, the author claim to invent a novel generative framework including a conditioned variational autoencoder and a diffusion model for designing antimicrobial peptides with specific physicochemical properties for specified target bacteria. Desgning good working antimicrobial peptides is a highly relevant question to overcome the more and more problematic antimicrobial resistance, we are facing world-wide. Finding an efficient framework to find new ones with specific properties is one of the key goals in this context.

Are these claims novel? If not, which published articles weaken the claims of originality of this one are the claims properly placed in the context of the previous literature? Have the authors treated the literature fairly?

- I am working in the field of the evolution and combatting of antimicrobial resistance, but I am not an expert in generating AMPs with methods like autoencoders or diffusion models. From their introduction it becomes clear what the current short-comes of the existent methods are and how their framework can fill them, but I am not really in the position to judge if there are important pieces of literature missing or of there is something out there making the same claims. I think here, the opinion from other reviewers or people in the field would be more improtant than my assessment.

Do the data and analyses fully support their claims? If not, what other evidenceis required?

- The authors claim that the advantage of pretrained datasets is visible from Figure S3. I can not really see directly that definitely is the case. Maybe there would some explanation be helpful, how this supports their claim?
- Isn’t the LLM based model giving even lower MIC ratios?
- In Table 2, I think it would be an important information on what targets the values from the other models come from. Looking only at the two models from the author, it seems to play an important factor. The E.coli model seems indeed to outperform the other models, but not so the one for S.aureus. So what if all others are also based on S.aureus, then that would not give any evidence that their model performce better.
- They state that from Figure 5E it is clearly visible that the distribution of generatied AMPs is between those of positive and negative samples (what is correct), indicating that they can generate AMPs with high antibacterial activity. But I was wondering if for high antibacterial activity, shouldn’t the distribution then be more like the one of the positive samples?
- All other shown results look like this could be a very promising new approach

Detailed protocols and algorithms?Original data available?

- There is a github page with the code available.
- The training data set cannot be uploaded there because it exceeds 100 MB, but they state there that it is available after request.
- I think the used software packages should also be cited in the paper
- The authors provide a step-for step guide how you can reproduce their pipeline
- The repository could be improved by extending the read me a bit and describe the folder structures or the additional data and scripts that are there. It feels there is a lot of useful and interesting material out there, but it is hard to get an overview what file contains what and how they relate. Also what do I need to change in the pipline, if I want to use it for example with other data?

Are details of the methodology sufficient to allow experiments to be reproduced

- What was the selection process of obtaining the 572619 proteins from UniProt? Which ones are in this set and why exactly these?

Is the manuscript well organized and written clearly enough to be accessible to non-specialists?

- The general structure, as well as the objective and the experiments, are presented in logical order and in a comprehensible manner
- My main concern here is that the Materials and Methods section requires that the reader is already familiar with the technical details of Conditional Variational Autoencoders and Diffusion Models. I am not a specialist in these topics and for me it was very hart and in parts even impossible to follow these sections, what shrinks the audience to people that work exactly with these models. I think it would be great, if this could be made a bit more accessible, maybe by adding some basic informations, or provide an attachement where you can get the necessary understanding.
- For example:
    - what is the basic underlying structure and mode of action of these models?
    - What is the prior transformer-based architecture they adopt in their framework?
    - what does it mean to replace the fully connected layers with convolutional operations?
    - what is a normalization procedure of the input attributes or dedicated fusion layers?
    - how are the loss functions integrated in the process ?
    - How does the embedding of an attribute encoder within the decoder enable gradient propagation of the loss?
    - What is a BERT-Encoder?
    - Where is the Markov Chain in subsection 2.3.2 coming from or how does it relate to the model?
- For me it is also not clearly visible what technical details differ from the current methods that make this framework better. I think that might be visible, when you are working in this field, but hard to see as a non-specialist.

Does the paper use standardized scientific nomenclature and abbreviations? If not, are these explained at the first usage?

- In the introduction there are shortenigs GAN and VAE without introducing them
- in the loss function on p.7: What are sigma_i and mu_i. I think you can guess that when you are familiar with VAEs, but I think it does no harm to shortly define or mention it here
- in the description of the prediction process (x_pred_t): what is f, what is theta and what is t?
- x_0 is one times defined as initial embedding representation of the data (p.9) and one time as the original data (p.7). Is that the same?
- In Figure 3 and the corresponding subsection 2.4.1: What are ESM and MLP?
- What is MSE (subsection 2.4.2)
- In the loss function (6), what is N?
- In the algorithm what are alpha_t, beta_t and sigma_t? What is f_theta^mean and g_phi ^logvar?

Figures:

- generally would some more detailed description in the captions itself be helpful. Many figures are understandable, when reading the whole text, but not when looking at the figure alone.
- In Figure 1A: What is Seq \oplus P in the first step?
- In Figure S5: What is Total Loss, Rec loss and PP loss?
- Table 1 presents values for different masses of target bacteria. Which ones are used in Figure 4?
- Figure 4C and D: I was wondering if there is a better way to visualise these data. Personally, I found i very hard to compare the different values from that.
- In Figure 5 I was not sure what I can see and what the different shapes mean. Also what do the red boxes indicate? But I think this can be fixed easily with a more detailed description in the caption.

- Also in Table 4: It would improve the accessability when you just give a short description in the caption which ones belong to E.coli and which ones to S. aureas, as you did in the text
