# Supplementary material for: A novel generative framework for designing pathogen-targeted antimicrobial peptides with programmable physicochemical properties
Source: PLoS Comput Biol. 2025 Dec 29;21(12):e1013833. doi: 10.1371/journal.pcbi.1013833 (PMC12747415; doi:10.1371/journal.pcbi.1013833)
Supplement: Attachment — Submitted filename: Review_PCOMPBIOL-D-25-01993.pdf [file pcbi.1013833.s008.pdf]

Summary: This manuscript presents a dual-function generative framework that integrates a CVAE and a conditional diffusion model to design antimicrobial peptides (AMPs) with both controllable physicochemical properties and pathogen specificity (E. coli and S. aureus). The authors pre-train on millions of UniProt-derived sequences, fine-tune using GRAMPA MIC-labeled data, and introduce an attribute preservation loss with an auxiliary predictor to enforce property control. A BERT-based diffusion model is used for conditional generation, and the framework is further supported by a bacteria-specific MIC predictor. The model outperforms several recent baselines in MIC-based activity and demonstrates strong control over multiple attributes. Finally, large-scale in silico screening, toxicity/hemolysis prediction, and membrane docking identify four highly promising AMP candidates with strong therapeutic potential.

Comments:

- **Loss function lacks weighting coefficients:**

In section 2.2.2. and Eq.1., the total loss combines KL divergence, reconstruction loss, and attribute preservation loss without any weighting factors. In practice, these terms differ in scale and importance; the absence of weighting or explanation raises concerns about training stability and reproducibility.

- **Insufficient architectural details of key modules (BERT, fusion, condition embedding):**

The manuscript does not provide adequate information about the BERT-based diffusion model (model size, pre-training, integration with condition vector) or the fusion layers in the CVAE. These omissions limit reproducibility.

- **Fairness of MIC predictor comparison is questionable:**

The authors' MIC predictor is bacteria-specific, whereas the baseline predictors (Table 2) appear to be generic. This specialization likely provides an inherent advantage and should be either justified explicitly or controlled by training baseline models per bacterium.

- **Limited assessment of novelty and diversity of generated peptides:**

While biological activity is evaluated, the manuscript does not report sequence similarity metrics, novelty percentages, or diversity analyses. Without these, it is unclear whether the model generates substantially new peptides or variants of known sequences.

- **Generalization beyond two bacterial species is untested:**

The framework is claimed to be broadly applicable, but only E. coli and S. aureus are used. It remains unclear whether the model can generalize to other pathogens or requires re-training from scratch.

- **Terminology consistency:**

Terms such as “attribute,” “property,” and “feature” are used interchangeably. Consistent terminology would improve clarity.

- **Details of t-SNE visualization missing:**

Hyperparameters (e.g., perplexity, number of iterations) are not reported, which affects interpretability of the latent space plots.

- **Segmentation procedure for UniProt sequences not fully described:**

The method of segmenting proteins into short sequences (e.g., stride, overlap) should be specified.
