# Supplementary material for: A novel generative framework for designing pathogen-targeted antimicrobial peptides with programmable physicochemical properties
Source: PLoS Comput Biol. 2025 Dec 29;21(12):e1013833. doi: 10.1371/journal.pcbi.1013833 (PMC12747415; doi:10.1371/journal.pcbi.1013833)
Supplement: Attachment — Submitted filename: Summary-of-Revision-1st-v1.3.pdf [file pcbi.1013833.s009.pdf]

Summary of Revisions

Title: A Novel Generative Framework for Designing Pathogen-targeted Antimicrobial Peptides with Programmable Physicochemical Properties

Authors: Weizhong Zhao, Kaijieyi Hou, Chang Tang, Yiting Shen, Jinlin Liu, Xiaohua Hu

We would like to thank all anonymous reviewers and editors for their valuable comments. They have helped to improve the paper in a number of ways. According to the comments, we have revised the manuscript carefully and highlighted revisions in the resubmitted manuscript. Specifically, the detailed responses to specific comments are listed as follows.

Response to Editor

Comment 1: Please ensure that the CRediT author contributions listed for every co-author are completed accurately and in full. At this stage, the following Authors/Authors require contributions: Weizhong Zhao, Xiaohua Hu, Yiting Shen, Kaijieyi Hou, and Jinlin Liu. Please ensure that the full contributions of each author are acknowledged in the "Add/Edit/Remove Authors" section of our submission form. The list of CRediT author contributions may be found here: https://journals.plos.org/ploscompbiol/s/authorship#loc-author-contributions
Response: Thanks for your comments. Following your comments, we have acknowledged the authors' contribution in the submission system. It is worth noting that a new author Chang Tang was involved in this study, in which his main contribution is to conduct the experiments of novelty analysis of our model and baselines. In addition, Chang Tang also helped to validate the results of other experiments, and to review and edit the revised manuscript. Thanks again for your valuable comments.

Comment 2: We ask that a manuscript source file is provided at Revision. Please upload your manuscript file as a .doc, .docx, .rtf or .tex. If you are providing a .tex file, please upload it under the item type u2018LaTeX Source Fileu2019 and leave your .pdf version as the item type u2018Manuscriptu2019.
Response: Thanks for your comments. The required versions of the source tex file and the pdf file have been uploaded in the submission system.

Comment 3: Please provide an Author Summary. This should appear in your manuscript between the Abstract (if applicable) and the Introduction, and should be 150-200 words long. The aim should be to make your findings accessible to a wide audience that includes both scientists and non-scientists. Sample summaries can be found on our website under Submission Guidelines:
https://journals.plos.org/ploscompbiol/s/submission-guidelines#loc-parts-of-a-submission
Response: Thanks for your comments. The Author Summary has been added following the

requirements and highlighted in the revised manuscript.

Comment 4: Your manuscript is missing the following section heading: Abstract. Please ensure all required sections are present and in the correct order. Make sure section heading levels are clearly indicated in the manuscript text, and limit sub-sections to 3 heading levels. An outline of the required sections can be consulted in our submission guidelines here: https://journals.plos.org/ploscompbiol/s/submission-guidelines#loc-parts-of-a-submission
Response: Thank you for your comments. We are sorry for the issue. Due to the problem with the LaTeX format, the Abstract title was not explicitly displayed in the previous manuscript. This issue has been addressed in the revised version.

Comment 5: Please upload all main figures as separate Figure files in .tif or .eps format. For more information about how to convert and format your figure files please see our guidelines: https://journals.plos.org/ploscompbiol/s/figures
Response: Thanks for your comments. All main figures were uploaded in .eps format. Note that all main figures have also been processed by the PLOS's NAAS tool to generate corresponding tif figure files, and confirmed that the figure files meet the journal's requirements.

Comment 6: Please revise your current Competing Interest statement to the standard "The authors have declared that no competing interests exist."
Response: Thank you for your comments. We have made the necessary changes in the corresponding section in the resubmitted manuscript.

Comment 7: We note that the Data Availability Statement mentioned in the manuscript is different from that provided in the online submission form. The Data Availability statement in the online submission form is currently as follows: 'All relevant data are within the manuscript and its Supporting Information files.' While the one in the manuscript states 'The data and source codes are available in GitHub at https://github.com/David-WZhao/AMPGen.' Please provide the complete Data Availability statement in the submission form and ensure that it matches the one mentioned in the manuscript.
Response: Thanks for pointing this issue. Following your comments, the issue has been addressed, i.e., the complete Data Availability statement in the submission form is matching the one mentioned in the manuscript.

Response to Reviewer #1
Comment 1: This is an interesting and well-written study that introduces a new deep learning framework to design pathogen-targeted AMPs with programmable physicochemical properties. The combination of a CVAE with a diffusion model is technically sound, and the results are promising. The manuscript is clearly within scope and generally well organized, but several areas could be improved to make the work more accessible and convincing.
Response: Thank you for your comments. We have made revisions to the manuscript based on the suggestions provided.

Comment 2: The authors should add a short paragraph that explicitly claim the novelty of this paper.
Response: Thank you for your comments. According to your suggestion, we have summarized the novelty of this paper and added it to the end of the Introduction section. The revisions have been highlighted in the resubmitted manuscript.

Comment 3: The authors rely on predicted MICs, toxicity scores, and docking simulations, which can all be biased by the datasets used to train those predictors. A short discussion paragraph on these in-silico limitations should be added in the discussion section.
Response: Thank you for constructive comments. Following your comments, we have revised the Discussion section to explicitly acknowledge this limitation. We now state that these in silico scores should be interpreted as heuristic prioritization rather than definitive evidence of efficacy or safety, and that all generated peptide candidates will require systematic experimental validation in future work. The corresponding revisions have been highlighted in the Discussion section in the revised manuscript.

Comment 4: In section 2.1.2, The criteria for “positive” vs. “negative” MIC thresholds (<10 μM and >20 μM) should be justified or referenced.
Response: Thank you for your valuable comments. We have revised section 2.1.2 to clarify the rationale behind the MIC thresholds. Specifically, peptides with MIC < 10 μM were labeled as positive (highly active) and peptides with MIC > 20 μM were labeled as negative (weakly active), because lower MIC indicates stronger antibacterial potency. Peptides with intermediate MIC values were excluded to avoid ambiguous borderline cases and to obtain two activity classes with comparable sample sizes for stable discriminative training. The selected MIC thresholds (<10 μM and >20 μM) were widely used in previous studies. Therefore, we employed these two thresholds as well in this study. We have added and highlighted the explanation and the corresponding references in section 2.1.2 in the revised manuscript.

Comment 5: In Section 3.2 or Section 3.3, the authors should include a simple measure of novelty (maybe average percent identity of generated peptides to their nearest training sequence or n-gram overlap?). This will confirm the model produces new AMPs rather than variants of known ones.
Response: Thank you for your valuable suggestion. We agree that introducing a simple measure of novelty would help confirm that our model generates truly new AMPs. To address this, we have added a novelty analysis based on n-gram (n=6, n=4) overlap. The results show that the peptides generated by our model exhibit high novelty. The detailed results and analysis have been added and highlighted in Section 3.2 of the revised manuscript.

Comment 6: In Figure 5D, why does increasing alpha-helicity and aromaticity boost antimicrobial activity? A brief biological rationale should be added explaining this trend.
Response: Thank you for your comments. We are sorry for the confusion. In the revised version, we have added a brief biological explanation for the settings in Fig. 5E (originally Fig. 5D) regarding increased alpha-helix and aromaticity, along with the corresponding references. In addition, the generated results support that appropriately increasing these two properties can enhance antimicrobial activity (Table 4).

Comment 7: In Section 3.3, the authors state isoelectric point behaves differently from other properties. The authors should include a short interpretation to show that they have considered this anomaly.

Response: Thank you for your valuable comments. We have revised Section 3.3 to include an interpretation of why the isoelectric point (pI) behaves differently from the other properties. More specifically, pI in short peptides is largely determined by the balance between basic (positively charged) and acidic (negatively charged) residues. Small changes in this balance can cause abrupt jumps in pI rather than smooth shifts. As a result, the generated peptides span a broad and relatively even pI range instead of collapsing toward extremely high pI. We also note that AMPs are typically cationic, but their activity depends on an overall charge balance rather than unbounded positive charge, which further explains why pI does not show the same directional bias as other properties. The detailed explanation has been added and highlighted in Section 3.3 of the revised manuscript. Thanks again for your constructive comments.

Comment 8: In Section 2.3.1, the text introduces “alpha” in Equation 2 as part of the noise schedule, but it is not linked to the later use of “alpha_t” and “alpha_(t−1)” in Algorithm 1. In addition, “beta_t” and “sigma_t” first appear in Algorithm 1 (Section 2.6) without any prior definition. The authors should explicitly define these schedules and parameters.

Response: Thank you for your valuable comments. According to your comments, we have revised the corresponding parts in Eq. 2 and Algorithm 1, and we have added a brief description of these variables as well in the resubmitted manuscript.

Comment 9: Replace “AMPs treating E. coli” with “AMPs targeting E. coli” throughout

Response: Thank you for pointing out this issue. We have gone through the whole manuscript and replaced “AMPs treatment E. coli” with “AMPs targeting E. coli” accordingly.

Comment 10: For Figures 4 and 5, add the number of generated peptides per condition and a short description of what each panel compares.

Response: Thank you for your valuable comments. We have clarified this part in the revised manuscript. More specifically, for both Fig. 4 and Fig. 5 each model generates 512 peptide sequences for each target condition/property. We have also added more detailed captions to describe the results in each panel.

Comment 11: For Figures S8 and S9, briefly restate what docking and confidence scores mean.

Response: Thank you for your comments. In the Supplementary Materials, we have updated the captions of Fig. S8 and S9 to briefly restate the meaning of the docking score and the confidence score.

Response to Reviewer #2

Comment 1: What are the main claims of the paper and how significant are they for the discipline?

• In this paper, the author claim to invent a novel generative framework including a conditioned variational autoencoder and a diffusion model for designing antimicrobial peptides with specific physicochemical properties for specified target bacteria. Desgning good working antimicrobial

peptides is a highly relevant question to overcome the more andmore problematic antimicrobial resistance, we are facing world-wide. Finding an efficient framework to find new ones with specific properties is one of the key goals in this context.
Response: Thank you for your comments.

Comment 2: Are these claims novel? If not, which published articles weaken the claims of originality of this one are the claims properly placed in the context of the previous literature? Have the authors treated the literature fairly?
• I am working in the field of the evolution and combatting of antimicrobial resistance, but I am not an expert in generating AMPs with methods like autoencoders or diffusion models. From their introduction it becomes clear what the current short-comes of the existent methods are and how their framework can fill them, but I am not really in the position to judge if there are important pieces of literature missing or of there is something out there making the same claims. I think here, the opinion from other reviewers or people in the field would be more important than my assessment.
Response: Thank you for your comments.

Comment 3: Do the data and analyses fully support their claims? If not, what other evidence is required?
• The authors claim that the advantage of pretrained datasets is visible from Figure S3. I can not really see directly that definitely is the case. Maybe there would some explanation be helpful, how this supports their claim?
Response: Thank you for your comments. We apologize the figure caption was not clear enough. Fig. S3 demonstrates the property retention of sequences generated by both pretrained and non-pretrained models by visualizing their distribution after dimensionality reduction. As shown in Fig. S3, the pretrained model exhibits a significantly higher property retention rate of 91.2% (with a mean squared error (MSE) threshold of < 0.0625), compared to just 68.2% for the non-pretrained model, and the distribution for the non-pretrained model is noticeably wider. This result highlights the critical advantage of pretraining. By leveraging a broader sequence space during pretraining, the model is better able to preserve essential biochemical and physicochemical properties during sequence generation. This property retention is crucial for generating functional AMPs with the desired characteristics.

Comment:• Isn’t the LLM based model giving even lower MIC ratios?
Response: Thank you for your comments. As shown in Table 1, our model generates a higher proportion of low MIC peptides compared to the LLM-based model. Specifically, for E. coli, the ratio of peptides with MIC values below 20 μM is 0.6508, and for S. aureus, the ratio is 0.85. Both values are higher than the corresponding ratios of 0.6252 and 0.7559 achieved by the LLM-based model, indicating that our model outperforms the LLM-based approach in generating peptides with lower MIC values.

Comment:• In Table 2, I think it would be an important information on what targets the values from the other models come from. Looking only at the two models from the author, it seems to play an important factor. The E. coli model seems indeed to outperform the other models, but not so the one for S.aureus. So what if all others are also based on S.aureus, then that would not give any

evidence that their model performance better.

Response: Thank you for your insightful comments. We agree that understanding the target organism for each model is critical for fair comparison. In our revised experiments, we addressed this by retraining all baseline models separately for each bacterium. Specifically, for both E. coli and S. aureus, we trained and evaluated our MIC predictor and the baseline models using the same data source (GRAMPA) and under identical settings for each organism. This means that all models are now bacterium-specific, and we have ensured that the training and testing data are consistent for each bacterium. This controlled setup eliminates any potential bias from differences in target organisms, allowing the performance differences to reflect the models' ability to predict MIC for each respective bacterium.

Specifically, the results show that our model performs better than or comparable to the baseline models. Notably, for predicting the MIC of AMPs against E. coli, our model achieves the best MSE and MAE. Although on S. aureus our metrics are slightly lower than those of AMP-MIC, the difference in MSE is only 0.008, and our model already shows a substantial improvement over the RNN-based and GPT-based baselines. This further confirms the effectiveness of our MIC predictor. The detailed results and analysis have been provided in Section 3.2 of the revised manuscript. Hope we address your concern.

Comment:• They state that from Figure 5E it is clearly visible that the distribution of generated AMPs is between those of positive and negative samples (what is correct), indicating that they can generate AMPs with high antibacterial activity. But I was wondering if for high antibacterial activity, shouldn’t the distribution then be more like the one of the positive samples?

Response: Thank you for your insightful comments. You are totally correct that for high antibacterial activity, the distribution of generated AMPs should be closer to that of the positive samples. However, the distribution of the generated AMPs observed in Fig. 5C (originally Fig. 5E), which falls between the positive and negative samples, may reflect a characteristic of the current generation process of the model. Without additional filtering or selection steps, the model generates a diverse range of peptides, including those with both high and low antibacterial activity. Nevertheless, the model still tends to generate peptides closer to the positive samples. This explains why the distribution spans between the positive and negative samples.

Comment:• All other shown results look like this could be a very promising new approach

Response: Thank you very much for your positive feedback.

Comment 4: Detailed protocols and algorithms? Original data available?

• There is a github page with the code available.

• The training data set cannot be uploaded there because it exceeds 100 MB, but they state there that it is available after request.

• I think the used software packages should also be cited in the paper

• The authors provide a step-for step guide how you can reproduce their pipeline

•The repository could be improved by extending the read me a bit and describe the folder structures or the additional data and scripts that are there. It feels there is a lot of useful and interesting material out there, but it is hard to get an overview what file contains what and how they relate. Also what do I need to change in the pipeline, if I want to use it for example with other data?

Response: Thank you for your suggestions. We have revised the README.md to include a more detailed description, including how to replace the dataset with your own.

Comment 5: Are details of the methodology sufficient to allow experiments to be reproduced

• What was the selection process of obtaining the 572619 proteins from UniProt? Which ones are in this set and why exactly these?

Response: Thank you for your comments. In this study, we used the reviewed version of the UniProt database (in 12/17/2024 with the source file entitled "uniprotkb_reviewed_true_2024_12_17.fasta") to ensure the high curation quality of the training data, avoiding potential redundancy from unreviewed (TrEMBL) entries. The selected dataset consists of proteins that have been reviewed and curated for accuracy, ensuring the reliability of the data used in training. Details on dataset processing have been added to Section 2.1.1 of the revised manuscript.

Comment 6: Is the manuscript well organized and written clearly enough to be accessible to non-specialists?

• The general structure, as well as the objective and the experiments, are presented in logical order and in a comprehensible manner

• My main concern here is that the Materials and Methods section requires that the reader is already familiar with the technical details of Conditional Variational Autoencoders and Diffusion Models. I am not a specialist in these topics and for me it was very hard and in parts even impossible to follow these sections, what shrinks the audience to people that work exactly with these models. I think it would be great, if this could be made a bit more accessible, maybe by adding some basic informations, or provide an attachement where you can get the necessary understanding.

• For example:

◦ what is the basic underlying structure and mode of action of these models?

◦ What is the prior transformer-based architecture they adopt in their framework?

◦ what does it mean to replace the fully connected layers with convolutional operations?

◦ what is a normalization procedure of the input attributes or dedicated fusion layers?

◦ how are the loss functions integrated in the process ?

◦ How does the embedding of an attribute encoder within the decoder enable gradient propagation of the loss?

◦ What is a BERT-Encoder?

◦ Where is the Markov Chain in subsection 2.3.2 coming from or how does it relate to the model?

• For me it is also not clearly visible what technical details differ from the current methods that make this framework better. I think that might be visible, when you are working in this field, but hard to see as a non-specialist.

Response: Thank you for your thoughtful comments. We understand that the Methods section may be challenging for non-specialists, especially regarding the technical details of Conditional Variational Autoencoders (CVAE) and Diffusion Models. To address this, we have added a dedicated section entitled "Details of Model" in the Supplementary Materials that provides a detailed explanation of the model architecture, including the CVAE's Encoder, latent space, Decoder, as well as the denoising module in the diffusion model and the conditional embedding module. This section is intended to provide a clearer understanding of the core components and methodology behind our

framework. We hope that this additional information helps clarify the technical aspects and makes the content more accessible to a broader audience. Thanks again for your valuable comments. Hope we address your concern.

Comment 7: Does the paper use standardized scientific nomenclature and abbreviations? If not, are these explained at the first usage?

• In the introduction there are shortenigs GAN and VAE without introducing them

Response: Thank you for your suggestion. We have already addressed this issue in the revised manuscript, i.e., providing the full name at its first occurrence in the manuscript.

Comment: • in the loss function on p.7: What are sigma_i and mu_i. I think you can guess that when you are familiar with VAEs, but I think it does no harm to shortly define or mention it here

Response: Thank you for your constructive comments. In the revised manuscript, we have added an explanation at the corresponding part to make it more accessible for a broader audience.

Comment: • in the description of the prediction process (x_pred_t): what is f, what is theta and what is t?

Response: Thank you for your comments. In the revised manuscript, we have added an explanation at the corresponding part to make it more accessible for a broader audience.

Comment: • x_0 is one times defined as initial embedding representation of the data (p.9) and one time as the original data (p.7). Is that the same?

Response: Thank you for your thoughtful comments. We are sorry for the confusion. Maybe we didn't describe it clearly in the previous version. x_0 refers to the same concept in both instances. It represents the initial embedding of the data in the diffusion model. One is used during the noise addition process, and the other is used in the denoising process for the loss function calculation. Please note that in calculation of the loss function, what is actually used is the linear combination of x_0 and noise. We have modified the corresponding Eq. 5. Thanks again for your valuable comments. Hope we address your concern.

Comment: • In Figure 3 and the corresponding subsection 2.4.1: What are ESM and MLP?

Response: Thank you for your comments. ESM stands for "Evolutionary Scale Modeling", which refers to a type of model used for protein sequence analysis, while MLP stands for "Multi-Layer Perceptron", a class of neural networks commonly used for various tasks, including classification and regression. We hope this clears up the terms used in Fig. 3 and subsection 2.4.1.

Comment: • What is MSE (subsection 2.4.2)

Response: Thank you for your question. MSE stands for "Mean Squared Error", which is a commonly used loss function in machine learning and statistics. It measures the average of the squared differences between the predicted values and the actual values, helping to quantify the error in the model's predictions.

Comment: • In the loss function (6), what is N?

Response: Thank you for your valuable comments. In the revised manuscript, we have added the

explanation for N in the corresponding section of the formula. Actually, it simply represents the index of the sequence. Hope we address your concern.

Comment: • In the algorithm what are alpha_t, beta_t and sigma_t? What is f_theta^mean and g_phi ^logvar?
Response: Thank you for your comments. In the revised manuscript, we have added a Notation section in Algorithm 1, where we provide explanations for the key parameters.

Comment 8: Figures:
• generally would some more detailed description in the captions itself be helpful. Many figures are understandable, when reading the whole text, but not when looking at the figure alone.
Response: Thank you for your constructive comments. In the revised manuscript, we have revised and expanded the captions to provide more detailed information, ensuring that the figures are clearer and more understandable even when viewed on their own.

Comment: • In Figure 1A: What is Seq \oplus P in the first step?
Response: Thank you for your question. The symbol $\oplus$ in the first step of Fig. 1A represents the operation of concatenation. We have added this explanation in the caption of the figure for clarification.

Comment: • In Figure S5: What is Total Loss, Rec loss and PP loss?
Response: Thank you for your question. We have added the corresponding explanations in the caption of Fig. S5. We apologize for any confusion this may have caused.

Comment: • Table 1 presents values for different masses of target bacteria. Which ones are used in Figure4?
Response: Thank you for your question. Fig. 4 presents both types of targeted AMPs. Specifically, Fig. 4A corresponds to the first column of Table 1, while Fig. 4B corresponds to the second column of Table 1. In other words, the quantitative data from Table 1 are visualized in Fig. 4. Thanks again for your valuable comments.

Comment: • Figure 4C and D: I was wondering if there is a better way to visualise these data. Personally, I found i very hard to compare the different values from that.
Response: Thank you for your suggestion. The data shown in Fig. 4C and D correspond to the quantitative values in Table 3. To ensure the fairness of the MIC predictor, we have redesigned the comparison experiment and presented the results using bar charts, which will make the comparison more straightforward and clearer. Thanks again for your constructive comments.

Comment: • In Figure 5 I was not sure what I can see and what the different shapes mean. Also what do the red boxes indicate? But I think this can be fixed easily with a more detailed description in the caption.
Response: Thank you for your comments. We have added a more detailed explanation in the caption of Fig. 5 to clarify what is shown and the meaning of the different shapes, as well as the indication of the red boxes. We hope this resolves the confusion.

Comment: • Also in Table 4: It would improve the accessability when you just give a short description in the caption which ones belong to E.coli and which ones to S. aureas, as you did in the text

Response: Thank you for your suggestion. In the revised manuscript, we have added a column to Table 5 (formerly Table 4) to indicate which bacteria are targeted.

Response to Reviewer #3

Comment 1: Loss function lacks weighting coefficients: In section 2.2.2. and Eq.1., the total loss combines KL divergence, reconstruction loss, and attribute preservation loss without any weighting factors. In practice, these terms differ in scale and importance; the absence of weighting or explanation raises concerns about training stability and reproducibility.

Response: Thank you for pointing this issue. We have updated Eq. 1 in the revised manuscript to clarify the weighting of the different loss terms. Specifically, we introduce an explicit coefficient beta on the KL divergence term, which is annealed during training (starting from a small value and increasing to its final value) to ensure training stability. The reconstruction loss and the property-preservation loss are kept with unit weight because both terms are on comparable scales after normalization of the target properties. We have also added this explanation to the text to make the optimization setup and reproducibility assumptions explicit. The specific functional form is implemented in the KLAnnealer class in “TransVAE.py”. Thanks again for your constructive comments. Hope we address your concern.

Comment 2: Insufficient architectural details of key modules (BERT, fusion, condition embedding): The manuscript does not provide adequate information about the BERT-based diffusion model (model size, pre-training, integration with condition vector) or the fusion layers in the CVAE. These omissions limit reproducibility.

Response: Thank you for your valuable comments. In the revised manuscript, we have added detailed architectural descriptions in Appendix A of the Supplementary Materials. Appendix A now includes: (i) the encoder, latent space and decoder design of the CVAE, (ii) the core components of the diffusion model, and (iii) key model hyperparameters. These additions are intended to improve the reproducibility. Hope we address your concern.

Comment 3: Fairness of MIC predictor comparison is questionable: The authors’ MIC predictor is bacteria-specific, whereas the baseline predictors (Table 2) appear to be generic. This specialization likely provides an inherent advantage and should be either justified explicitly or controlled by training baseline models per bacterium.

Response: We appreciate the reviewer’s concern regarding fairness in the MIC predictor comparison. In the revised experiments, we addressed this by retraining all baseline models separately for each bacterium. More specifically, for both E. coli and S. aureus, we trained and evaluated our MIC predictor and the baseline predictors under the same setting, using the same data source (GRAMPA) for each organism. In other words, all models are now bacterium-specific and are compared on matched training and testing splits for the same bacterium. This controlled setup

ensures that the reported performance differences are not due to specialization bias, but instead reflect the relative modeling ability under identical conditions.

Comment 4: Limited assessment of novelty and diversity of generated peptides: While biological activity is evaluated, the manuscript does not report sequence similarity metrics, novelty percentages, or diversity analyses. Without these, it is unclear whether the model generates substantially new peptides or variants of known sequences.
Response: Thank you for your valuable comments. We agree that assessing novelty and diversity is important to determine whether the model is generating genuinely new AMPs rather than minor variations of known sequences. In the revised manuscript, we have added a quantitative analysis of sequence novelty and diversity. Specifically, we evaluate n-gram (n=6 and n=4) overlap between generated peptides and known peptides to estimate sequence-level novelty. The results show very high novelty scores (6-gram = 0.9998 and 4-gram = 0.9484), indicating that our model is able to generate peptides with low overlap to existing sequences. We have also compared our model to several baselines and observed a clear advantage in terms of novelty. These analyses have been included in Section 3.2. We believe this directly addresses the concern by demonstrating that the model does not simply reproduce known AMPs, but is capable of generating diverse and previously unseen peptide candidates. Thanks again for your constructive comments.

Comment 5: Generalization beyond two bacterial species is untested: The framework is claimed to be broadly applicable, but only E. coli and S. aureus are used. It remains unclear whether the model can generalize to other pathogens or requires re-training from scratch.
Response: Thank you for your comments. We agree with the reviewer's concern. In this work, we selected E. coli (Gram-negative) and S. aureus (Gram-positive) because they are widely used as representative pathogens in AMP studies and cover two major bacterial classes of clinical relevance. Our goal was to demonstrate that the proposed framework is effective across both Gram-negative and Gram-positive bacteria, rather than optimize for a single species. We acknowledge that broader generalization to additional pathogens (e.g., P. multocida, S. enteritidis, etc.) is an important next step. This does not require training the model from scratch; rather, the same framework can be adapted to a new pathogen by fine-tuning on species-specific data. We have added this clarification and limitation to the Discussion section of the revised manuscript.

Comment 6: Terminology consistency: Terms such as "attribute," "property," and "feature" are used interchangeably. Consistent terminology would improve clarity.
Response: Thank you for your comments. We appreciate the reviewer's observation. We agree that the terms "attribute", "property", and "feature" were used interchangeably in earlier drafts. In the revised manuscript, we have standardized the terminology and now consistently refer to these quantities as "properties" in the whole manuscript.

Comment 7: Details of t-SNE visualization missing: Hyperparameters (e.g., perplexity, number of iterations) are not reported, which affects interpretability of the latent space plots.
Response: We appreciate the reviewer's comment. The t-SNE hyperparameters are: perplexity = 30 (default), number of iterations = 1000 (default) and a fixed random_state = 43. The same settings are used for both positive and negative samples. The detailed descriptions have been added to the

updated caption of Fig. 6 in the revised manuscript.

Comment 8: Segmentation procedure for UniProt sequences not fully described: The method of segmenting proteins into short sequences (e.g., stride, overlap) should be specified.
Response: Thank you for your comments. In our method, protein sequences from the UniProt database (in 12/17/2024 with the source file entitled “uniprotkb_reviewed_true_2024_12_17.fasta”) are segmented using a sliding window approach, where the sequences are divided into shorter segments ranging from 2 to 50 amino acids. The window size is randomly selected from this range, and overlapping segments are allowed as the window slides across the sequence. To ensure balance, we aim for an equal number of segments for each length by stopping the segmentation process once the target count for each length is reached. The detailed descriptions have been added to the revised manuscript for clarity.

Response to Reviewer #4

Comment 1: Zhao et al. introduces a two-stage generative framework for designing antimicrobial peptides (AMPs) that are tailored to specific pathogens with desirable physicochemical properties. The approaches creatively combine CVAE for sequence generation and conditional diffusion model for fine-tuned on specific pathogens, plus MIC predictor to evaluate antimicrobial efficacy. Overall, the work is well-organized, technically solid. However, several aspects could be improved.
Response: Thank you for your comments. We have made revisions to the manuscript based on the suggestions provided.

Comment 2: In 2.2.2. loss functions of CVAE, three items were combined into Eq.1. However, appears that no weights were applied to items. Did the authors use any weights for loss functions to balance the training? If yes, please report them; if not, please justify why equal weighting worked and how you tuned it.
Response: Thank you for your valuable feedback. According to your comments, we have updated Eq. 1 in the revised manuscript to provide more clarity on how the different loss terms are weighted. We have introduced a coefficient, beta, for the KL divergence term, which is annealed during training to enhance stability, gradually increasing from a small initial value to its final value. The reconstruction loss and property-preservation loss are assigned unit weight, as both are on comparable scales after normalizing the target properties. We have clarified this issue in the manuscript to ensure transparency in our optimization process and to make the assumptions related to reproducibility explicit. The functional implementation of this approach can be found in the KLAnnealer class in the TransVAE.py file. Hope we address your concern.

Comment 3: In 3.2 model evaluation: the evaluation uses the authors’ MIC predictor to score all models’ outputs. This is reasonable but also potentially introduces bias if the predictor has learned patterns more aligned with the proposed model’s outputs. The authors should clarify how baseline sequences were obtained and evaluated. In addition, they compare MIC predictor performance to others in Table 2, showing slightly better results in MSE/MAE than ESKAPEE-MIC. Could you explain why the performance are so similar and discuss what strategies could be utilized to improve

the performance.
Response: Thank you for your insightful comments. We understand your concern regarding the potential bias introduced by using the MIC predictor to score all models' outputs. To address this, we have improved our experiments by selecting baseline models from the LLM-based paper for comparison, where these predictor models are trained separately for different bacteria. Specifically, we trained and evaluated both our MIC predictor and the baseline models for E. coli and S. aureus under the same experimental conditions, using the same data source (GRAMPA) for each organism. This controlled setup eliminates any bias related to specialization and ensures that the reported performance differences accurately reflect the relative modeling abilities of the predictors. Regarding the comparison of MIC predictor performance, we observed that our model slightly outperforms AMP-MIC in terms of MSE/MAE, but the difference is relatively small. This suggests that, although our model shows slight improvements, there may be limited room for further performance enhancement within the current evaluation framework. To further improve performance, strategies such as incorporating additional feature engineering, exploring more advanced deep learning architectures, and other potential approaches could be considered as directions for future improvements. Thanks again for your constructive comments. Hope we address your concern.

Comment 4: I am wondering the novelty of star candidate AMPs designed by the framework. The authors may inspect their similarity with known AMPs.
Response: Thank you for your valuable comments. We agree that assessing the novelty of the star candidate AMPs is important to confirm that our model generates truly unique peptides. To address this, we have conducted a novelty analysis using n-gram (n=6 and n=4) overlap metrics. The results demonstrate that the peptides generated by our model exhibit high novelty, with minimal similarity to known AMPs. This analysis has been added to Section 3.2 of the revised manuscript.

Comment 5: In 2.5. "contrastive fine-tuning is performed…" what is "contrastive fine-tuning"? No contrastive objective is defined.
Response: Thank you for pointing out this issue. You are correct that the term "contrastive fine-tuning" is not accurately used in our manuscript. We apologize for the confusion. In fact, we performed a supervised discriminative training using two types of labels, rather than employing a contrastive learning approach. We have revised the corresponding descriptions in the resubmitted manuscript to clarify this point. This will help avoid any confusion and ensure the terminology is used correctly. Thanks again for your valuable comments.

Comment 6: how many sequences were generated per model for Fig. 4 and Table 1.
Response: Thank you for your comments. We have clarified this issue in the revised manuscript. Specifically, for both Fig. 4 and Table 1, each model generates 512 peptide sequences in our experiments.

Comment 7: Model: specify the system size, CPU/GPU counts and runtime for both per step and total.
Response: Thank you for the question. We have added a description of the computational setup and runtime to the revised manuscript. More specifically, all experiments were run on a single

workstation equipped with one NVIDIA RTX 3090 GPU (24 GB VRAM). No multi-node or large-cluster resources were required. We have added these system details to section 3.1.

For the CVAE model, training was performed on a single NVIDIA RTX 3090 (24 GB) GPU with a batch size of 512 sequences per step (3-layer Transformer encoder/decoder). A complete training run of 50 epochs finished within one day on this hardware. For the diffusion model, we trained the denoising network (3-layer Transformer-style denoiser) under the same single-GPU setup. Training for 200 epochs required only ten hours, and the fine-tuning stage on a new bacterial species required less than half an hour. This further demonstrates that adapting the model to additional bacteria can be done efficiently without multi-GPU or cluster resources. For the MIC predictor, which is a lighter-weight regression model trained separately for E. coli and S. aureus, and end-to-end training completed in under an hour per organism.

Comment 8: Figure 5: no y label. Add detailed description for each panel. In addition, please re-organize Fig 5 in the main text because Fig 5E occurs earlier than 5C.
Response: Thank you for your valuable comments. Following your suggestion, in the revised manuscript, we have added the missing *y*-axis label and expanded the panel descriptions in Fig. 5. We have also reorganized the figure panels in the main text so that their order matches the order of appearance in the narrative. Thanks again for your constructive comments.

Comment 9: Page 17. "Interestingly, regardless of how to set the targeted attribute of Isoelectric Point, this property distribution remains quite even, which may require further investigation." Please discuss the reasons in Discussion.
Response: Thank you for your comments. According to your comment, we have revised Section 3.3 to include an explanation of the differences between isoelectric point (pI) and the other properties.

Comment 10: Page 20. "This concentrated distribution allows the generative model to stably sample from this region, producing more high-antimicrobial activity AMPs with similar properties and functions. In contrast, the distribution of negative samples in the latent space exhibits greater dispersion. This suggests that the latent representation of negative samples is more complex, potentially related to diverse noise patterns or variations in features. Compared to the high-density clustering of positive samples,the more dispersed nature of negative samples presents a higher challenge for the generative model when treating AMPs with low antimicrobial activity. This difference further highlights the generative model's focus on the generation direction of positive samples, optimizing its ability to generate AMPs with high-antimicrobial activity." This paragraph should move to Discussion.
Response: Thank you for your suggestion. Following your comments, we have moved the corresponding paragraph to the Discussion section in the revised manuscript.

Comment 11: Fig 7. Legend. "In vitro test" should be changed to "In silico test" because only simulation experiments were performed.
Response: Thank you for your valuable suggestion. In the revised manuscript, we have replaced "In vitro test" with "In silico test" in Fig. 7.

Comment 12: Provide more methodological details about docking and MD simulations (e.g., systems construction, force field, running parameters and time).

Response: Thank you for your valuable comments. In this study, we performed docking analysis using a standard blind docking protocol with default settings. Specifically, peptide candidates were docked to the target without imposing predefined binding site constraints (i.e., an exhaustive search over the entire receptor surface), and we relied on the software's default scoring function and search parameters to obtain the highest-ranked poses. This design has two advantages: first, it does not depend on prior knowledge of binding sites, which better reflects realistic screening scenarios for novel peptides with unknown mechanisms of action; second, it provides a fair and reproducible comparison across all candidates by minimizing manual tuning of docking hyperparameters. We have added this explanation to Section 3.5 in the revised manuscript.

Response to Reviewer #5

Comment 1: This manuscript by Zhao and colleagues introduces an innovative generative framework for the design of antimicrobial peptides (AMPs) with tunable physicochemical properties and pathogen-specific targeting. The approach combines a conditional Variational Autoencoder (CVAE) with a conditional diffusion model, further supported by a MIC predictor. The framework is rigorously evaluated through simulation experiments, visual analyses, and in silico screening, demonstrating notable improvements over existing methods in generating potent, low-toxicity AMPs targeting E. coli and S. aureus.

Overall, the study presents a compelling and technically robust methodology with significant potential to advance antimicrobial drug discovery. With further experimental validation and expansion to a broader range of pathogens, this framework could become a foundational tool for next-generation AMP development. I have the following comments.

Response: Thank you for your comments. We have made revisions to the manuscript based on the suggestions provided.

Comment 2: The results presented are comprehensive and well-supported by in silico analyses, however, the manuscript does not mention any experimental validation (e.g., antimicrobial assays, hemolysis tests). Including wet-lab validation for at least one generated AMP would significantly enhance the translational relevance and practical impact of the study.

Response: Thank you for your valuable comments. We totally agree that experimental validation would further strengthen the translational impact of the work. At this stage, our study is purely computational: all evaluations were performed in silico. We currently do not have access to the experimental infrastructure required to perform standardized wet-lab assays on the generated peptides, and therefore did not include in vitro or in vivo measurements in this version of the work. We acknowledge that this is a limitation of this study. In the revised manuscript, we have added this point to the Discussion and explicitly stated that prospective wet-lab validation of top-ranked candidate peptides is a key next step toward translation. Thanks again for your valuable comments.

Comment 3: The model appears to be trained primarily on E. coli and S. aureus, with limited representation of other bacterial species. Expanding the training dataset to include a broader range

of pathogens, particularly non-ESKAPE organisms, would improve generalizability. Alternatively, a discussion of potential transfer learning strategies could be beneficial.

Response: Thank you for your valuable comments. We agree that broader pathogen coverage is essential for assessing the generalizability of the model. In this study, we focused on E. coli (Gram-negative) and S. aureus (Gram-positive), which are widely used reference organisms in antimicrobial peptide research and represent two clinically relevant bacterial classes. Our primary goal was to demonstrate that the proposed framework can be applied to both Gram-negative and Gram-positive bacteria, rather than being limited to a single species.

We recognize that extending the model to additional pathogens, including non-ESKAPE species, is an important next step. Importantly, this extension does not require retraining the entire model from scratch. The framework can be transferred to a new bacterium by fine-tuning on species-specific data. In addition, we have discussed potential limitations and challenges associated with this process in the Discussion section of the revised manuscript.

Comment 4: The manuscript does not address the framework's applicability to longer peptides or non-peptide antimicrobial agents. A brief discussion on the potential limitations or extensions regarding peptide length and structural diversity would help clarify the scope of the approach.

Response: Thank you for your valuable comments. In the current framework, both the CVAE and diffusion models operate on fixed-length amino acid sequences, and the sequence length upper bound can be adjusted as a hyperparameter to support longer peptides. In this study, we restrict the length to $\leqslant 50$ amino acid residues. This reflects our focus on short and medium-length antimicrobial peptides, which constitute the majority of experimentally characterized AMP candidates in publicly available datasets.

Moreover, our approach has good scalability: (i) in principle, it can be extended to longer peptides by increasing the maximum positional length and retraining or fine-tuning on longer sequences; and (ii) supporting non-peptide antimicrobial agents would require representing molecules in a different form (e.g., SMILES-based encoding), which is not directly handled by the current sequence-based peptide generator. These points have been added to the Discussion section to clarify the intended scope of our approach.

Comment 5: The model seems to struggle with controlling the isoelectric point of generated sequences. It may be helpful to investigate whether this limitation arises from the underlying data distribution, encoding strategy, or model architecture. Incorporating a regularization term or exploring alternative encoding methods could improve control over this property.

Response: Thank you for your insightful comments. We have revised Section 3.3 to clarify why the isoelectric point (pI) behaves differently from the other controlled properties. In particular, we now discuss that, for short peptides, pI is primarily determined by the balance between basic (positively charged) and acidic (negatively charged) residues. Because pI is highly sensitive to small changes in this balance, even a single residue substitution can produce an abrupt jump in pI rather than a smooth incremental change. Consequently, the generated peptides tend to cover a broad and relatively uniform range of pI values instead of collapsing toward extremely high pI.

We have also added an explanation that AMPs are generally cationic, but their activity relies on maintaining an appropriate net charge rather than maximizing positive charge without bound. This functional constraint helps prevent strong directional bias in pI compared to other properties.

The above discussion has been incorporated into Section 3.3 of the revised manuscript.

Comment 6: It is not entirely clear which core datasets from UniProt were used in this study. UniProt provides several distinct resources, including UniProtKB for protein sequences, Proteomes for species-level data, UniRef for protein clustering, and UniParc as a sequence archive. Additionally, UniProt distinguishes between reviewed (Swiss-Prot) and unreviewed (TrEMBL) entries, which vary in terms of curation and redundancy. Clarifying which subset was utilized, particularly whether reviewed or unreviewed data were included, would be helpful. If unreviewed data were used, please describe how redundancy and annotation quality were addressed. It would also be valuable to comment on whether the UniProt and GRAMPA datasets employed in this study adequately represent the diversity of real-world antimicrobial peptides across different species and environments.

Response: Thank you for your comments. In this study, we used the reviewed version of the UniProt database (in 12/17/2024 with the source file entitled "uniprotkb_reviewed_true_2024_12_17.fasta") to ensure that the training data had high curation quality, thereby avoiding the potential redundancy associated with unreviewed (TrEMBL) entries. Details on dataset processing have been added to Section 2.1.1 of the revised manuscript. The UniProt data was primarily used to pretrain the CVAE model, enabling the design of peptides with programmable physicochemical properties. On the other hand, the GRAMPA dataset, which includes MIC data for a variety of AMPs against different bacterial species, was used to fine-tune the conditional diffusion model, allowing it to sample high-quality AMP representations. While the GRAMPA datasets used in this study are comprehensive and cover a wide range of AMPs with associated MIC data for different bacterial species, incorporating additional peptides from diverse species and environmental contexts in future studies could further enhance the representativeness of real-world antimicrobial peptide diversity. Hope we address your concern.

Comment 7: The proposed framework demonstrates impressive performance, but AI-based generative models like the CVAE and diffusion components used here are inherently black-box systems. I encourage the authors to provide additional interpretability insights, such as feature attribution, latent space diagnostics, or decision rationale from the MIC predictor. This would enhance transparency and trust, especially for clinical or experimental applications where explainability is critical.

Response: Thank you for your valuable comments. We understand the importance of interpretability, especially in clinical and experimental applications where explainability is critical. In response to your comments, we have added further details on the model architecture in Section A of the Supplementary Materials. This section includes an in-depth description of the core modules of our framework, such as the Encoder, latent space, Decoder in the CVAE, and the denoising network, along with the condition embedding in the diffusion model. We believe that these additions will provide greater transparency and help address concerns regarding the black-box nature of the model, thus enhancing its interpretability.

Comment 8: While docking scores below –300 and confidence scores above 0.96 are promising, it would be helpful to include comparative benchmarks or reference peptides to contextualize these values.

Response: Thank you for your valuable comments. In Section 3.5 of main file and the figure captions for Fig. S8 and S9 in the supplementary materials, we have added docking scores and confidence benchmarks to provide clearer background information and avoid any potential confusion for the readers. Specifically, we clarified the following points: Docking Scores (from ITScorePP/ITScorePR): A smaller negative value indicates stronger predicted binding, with scores of -200 or lower considered as strong binding. Confidence Scores: A value above 0.7 indicates a high likelihood of binding, a value between 0.5 and 0.7 suggests a possible binding, and a value below 0.5 indicates a low likelihood of binding. Thanks again for your constructive comments. Hope we address your concern.

Comment 9: Consider summarizing key findings from Tables S2–S4 in a graphical format (e.g., radar plots or heatmaps) to enhance interpretability of attribute differences across AMP variants.
Response: Thank you for your suggestion. We are sorry for the confusion. Maybe we didn't describe this part clearly in the previous version. Tables S2–S4 are primarily included to ensure the reproducibility of the experiments, as they specify the target condition values used during the study. Specifically, Table S4 focuses on the optimization of the properties for Cecropin, and the rationale behind the selection of these properties has been elaborated in the fourth paragraph of Section 3.3. Therefore, visualizing these tables in a graphical format, such as radar plots or heatmaps, may not be the most appropriate way to convey this information, as their primary role is to define experimental parameters rather than represent attribute differences across AMP variants. Thanks again for your valuable comment. Hope we address your concern.

Comment 10: In the Introduction.
a. Provide the full form of GRAMPA?
b. The authors mentioned "we first sample the noise data"- Could they clarify what kind of noise is meant in this context?
Response: Thank you for your valuable suggestion. We have made the relevant revisions in the Introduction section. Specifically, we have added the full name of GRAMPA, which is the Giant Repository of AMP Activities, and provided a clarification regarding the "noise data" as being sampled from a standard normal distribution (Gaussian noise).

Comment 11: Ensure consistent formatting of units.
Response: Thank you for pointing out this issue. We have reviewed the manuscript and standardized the formatting of the variables in the section 2.3.1. Additionally, we have included explanations for some of the variables in Algorithm 1 to guarantee the clarity.

Comment 12: Clarify the role of the MIC predictor in generation vs. evaluation.
Response: Thank you for your comments. In our study, the MIC predictor plays distinct roles during the generation and evaluation phases. During the generation phase, the MIC predictor is not directly involved in generating the AMPs; rather, it is used in the evaluation phase to assess the predicted efficacy of the generated peptides against specific bacterial species. Specifically, once the peptides are generated by the framework, their MIC values are predicted by the MIC predictor to evaluate their antimicrobial potential. This helps in filtering and ranking the generated AMPs based on their predicted antimicrobial activity, ensuring that the most promising candidates are selected for further

analysis. We have clarified this distinction in Section 2.4.1 of the revised manuscript for better understanding.

Comment 13: Include more details on the docking scoring functions and thresholds used.
Response: Thank you for your comments. We have added additional details regarding the docking in Section 3.5 of the revised manuscript. Specifically, the docking score, which is a critical metric in this analysis, was calculated using the iterative scoring functions ITScorePP or ITScorePR. A more negative docking score indicates a higher likelihood of binding, with a score around -200 or lower being considered ideal. Additionally, a confidence score greater than 0.7 suggests a high probability of binding, a score between 0.5 and 0.7 indicates a possible binding interaction, and a score below 0.5 suggests a low likelihood of interaction. These thresholds have been clearly outlined in the manuscript to enhance transparency in the docking evaluation process.

Comment 14: To support reproducibility and facilitate further research, please consider including the link to your implementation in the abstract. For example: "The source code for our framework is available at https://github.com/David-WZhao/AMPGen." This would make it easier for readers to access and explore your work directly.
Response: Thank you for your suggestion. We have added the link to the source code in the abstract, which will make it easier for readers to directly access and explore our work.
