# Supplementary material for: A novel generative framework for designing pathogen-targeted antimicrobial peptides with programmable physicochemical properties
Source: PLoS Comput Biol. 2025 Dec 29;21(12):e1013833. doi: 10.1371/journal.pcbi.1013833 (PMC12747415; doi:10.1371/journal.pcbi.1013833)
Supplement: Attachment — Submitted filename: PCOMP.pdf [file pcbi.1013833.s010.pdf]

First, I would like to thank you for the revisions made so far, especially for adding the novelty analysis and for expanding the explanation of the segmentation procedure. However, there are still several important points that require clarification and further refinement in order to ensure full transparency.

Comment 1:
Regarding the novelty and diversity analysis, although the inclusion of Table 2 and the 6-gram and 4-gram novelty scores is appreciated, I strongly recommend presenting this part as a separate subsection so that the metrics and methodology can be described in a complete and self-contained manner. At the moment, the definition of the 6-gram and 4-gram overlap remains unclear. It is not specified what the overlap is being measured against: the reference AMP database?, the training set?, sequences generated by other models?, or some other source??. Without explicitly defining the reference set, the novelty values cannot be properly interpreted. Additionally, the manuscript does not indicate how many 6-grams and 4-grams were examined, how the overlap was normalized, or whether novelty is defined as 1 − overlap or based on another formulation. Because peptide sequences are relatively short, extremely high novelty values such as 0.9998 for 6-grams require a clear methodological explanation to confirm that the computation is sound.
Furthermore, diversity has not been evaluated, even though diversity is just as important as novelty in generative peptide research. Novelty measures how different generated peptides are from known sequences, whereas diversity measures how different the generated peptides are from one another. Without assessing diversity, it is impossible to determine whether the model is producing a wide range of structurally and physicochemically distinct peptides or merely generating small variations of a limited set of motifs. Diversity can be quantified using standard metrics such as sequence entropy, pairwise distance distributions, clustering-based diversity, or physicochemical diversity across generated samples. Including such analyses is essential, because high novelty alone can even result from generating random or biologically implausible sequences, which may not reflect true generative capability. For the comparisons in Table 2, additional methodological clarity is needed. It should be stated how many sequences each model generated, whether the sequence lengths were controlled and matched across methods, and whether the same sampling procedures were used. Without having these conditions specified, the comparisons may be difficult to interpret reliably.

Comment 2:
With respect to the segmentation procedure, I appreciate that additional explanation has been added, but several key details are still missing. In particular, the stride used for the sliding window has not been specified. It remains unclear whether the stride was fixed, equal to one, proportional to the window size, or randomly chosen. The stride directly affects the density of extracted fragments and the distribution of n-grams, so it must be clearly defined for the segmentation to be reproducible.
Similarly, the balancing strategy for ensuring an equal distribution of segment lengths has not been described with sufficient precision. The manuscript mentions stopping once a “target count” for each length is reached, but the basis for selecting these target counts is not explained. It is important to clarify whether these targets were derived from actual AMP length distributions, set uniformly across lengths, or chosen using another criterion. Additionally, since the window size is randomly selected between 2 and 50, there is a risk of introducing bias toward certain lengths unless this process is carefully controlled. The manuscript does not currently explain what mechanisms, if any, were used to prevent such bias or to ensure that shorter or longer fragments were not disproportionately represented.
