# Supplementary material for: A novel generative framework for designing pathogen-targeted antimicrobial peptides with programmable physicochemical properties
Source: PLoS Comput Biol. 2025 Dec 29;21(12):e1013833. doi: 10.1371/journal.pcbi.1013833 (PMC12747415; doi:10.1371/journal.pcbi.1013833)
Supplement: Attachment — Submitted filename: Summary-of-Revision-2st-v1.1.pdf [file pcbi.1013833.s011.pdf]

Summary of Revisions

Title: A Novel Generative Framework for Designing Pathogen-targeted Antimicrobial Peptides with Programmable Physicochemical Properties

Authors: Weizhong Zhao, Kaijieyi Hou, Chang Tang, Yiting Shen, Jinlin Liu, Xiaohua Hu

We would like to thank all anonymous reviewers and editors for their valuable comments. They have helped to improve the paper in a number of ways. According to the comments, we have revised the manuscript carefully and highlighted revisions in the resubmitted manuscript. Specifically, the detailed responses to specific comments are listed as follows.

Response to Reviewer #1

Comment: I thank the authors for revising the manuscript and addressing my comments. The revised manuscript is now suitable for publication.

Response: We sincerely appreciate your time and effort in reviewing our manuscript and for your positive assessment. We are grateful for your constructive comments and suggestions, which have helped us improve the quality and clarity of the paper. Thank you again for your recommendation and support.

Response to Reviewer #2

Comment 1: All of my comments from the first review have been addressed by the authors. Especially section A in the SI makes it much more accessible to a broader audience, that could be interested in it. There are only two minor suggestions, that I still have.

Response: Thank you for your comments. We sincerely thank you for taking the time and effort to review our manuscript and for providing a positive evaluation. We have carefully considered the two additional minor suggestions you proposed and have revised the manuscript accordingly. We believe that these revisions have further improved the clarity and readability of the paper.

Comment 2: The authors explained to me in their response what ESM and MLP (Fig 3 and subsection 2.4.1) means. I think it would improve this section, if you just include the full name of these abbreviations als in the figure caption or in the text

Response: Thank you for your valuable suggestion. In the revised version, we have added brief explanations of ESM and MLP in the caption of Fig. 3, and we have included the full name of MLP at its first occurrence in subsection 2.4.1. We hope that these revisions improve the readability and clarity of this section.

Comment 3: It is clear from the text what is shown in tables and figures. However, adding some of the information also in the captions (f.e. Table 4, that the numbers that are shown there are

proportions) could improve the accessibility even further.
Response: Thank you for this helpful suggestion. In the revised manuscript, we have added clarifying notes to the tables that might cause confusion, such as Table 1 and Table 4, explicitly indicating that the reported values are proportions. We believe these additions further improve the accessibility and readability of the results.

Comment 4: Overall, I think the quality of this article improved significantly, and it could provide an interesting and promising new approach.
Response: Thank you for your comments. We sincerely thank you for your positive assessment and are pleased to hear that you find the quality of the article significantly improved. We are also encouraged that you consider our method an interesting and promising new approach.

Response to Reviewer #3

Comment 1: First, I would like to thank you for the revisions made so far, especially for adding the novelty analysis and for expanding the explanation of the segmentation procedure. However, there are still several important points that require clarification and further refinement in order to ensure full transparency.
Response: We sincerely thank you for carefully reviewing our manuscript and for acknowledging the revisions we have made so far. We are very grateful for your constructive comments and fully agree that further clarification is needed to ensure complete transparency. We have carefully addressed all remaining issues and have refined the manuscript.

Comment 2.1: Regarding the novelty and diversity analysis, although the inclusion of Table 2 and the 6-gram and 4-gram novelty scores is appreciated, I strongly recommend presenting this part as a separate subsection so that the metrics and methodology can be described in a complete and self-contained manner. At the moment, the definition of the 6-gram and 4-gram overlap remains unclear. It is not specified what the overlap is being measured against: the reference AMP database?, the training set?, sequences generated by other models?, or some other source??. Without explicitly defining the reference set, the novelty values cannot be properly interpreted. Additionally, the manuscript does not indicate how many 6-grams and 4-grams were examined, how the overlap was normalized, or whether novelty is defined as 1 − overlap or based on another formulation. Because peptide sequences are relatively short, extremely high novelty values such as 0.9998 for 6-grams require a clear methodological explanation to confirm that the computation is sound.
Response: Thank you for pointing out this important issue regarding the definition of n-gram overlap and novelty. In our implementation, the reference set of n-gram is constructed from all experimentally validated AMPs in the GRAMPA database, which provides a high-quality, curated collection of AMPs. Specifically, we first merge all AMPs from GRAMPA into a single FASTA file and extract all contiguous 4-gram and 6-gram (using a sliding window with stride 1) from these sequences; the union of these n-gram forms the N_{ref}. For each set of generated peptides, we likewise extract all contiguous 4-gram or 6-gram and collect the unique n-gram into a set N_{new}. The novelty score is defined as:

$$novelty = 1 - \frac{|N_{new} \cap N_{ref}|}{|N_{new}|}$$

So that a value close to 1 indicates that only a very small proportion of n-grams in the generated peptides are already present in GRAMPA, whereas a value close to 0 would indicate that most *n*-grams are shared with the reference database.

The possible reason of the very high 6-gram novelty values (e.g. 0.9998) lies in that exact 6-gram matches are extremely sparse: only about 0.02% of the unique 6-grams in the generated peptides are also found in GRAMPA, which is consistent with the large combinatorial space of 6-residue patterns and the high diversity of our generated sequences. We have added these methodological details and the explicit formula for overlap and novelty to the revised manuscript to make the analysis fully transparent and reproducible.

Comment 2.2: Furthermore, diversity has not been evaluated, even though diversity is just as important as novelty in generative peptide research. Novelty measures how different generated peptides are from known sequences, whereas diversity measures how different the generated peptides are from one another. Without assessing diversity, it is impossible to determine whether the model is producing a wide range of structurally and physicochemically distinct peptides or merely generating small variations of a limited set of motifs. Diversity can be quantified using standard metrics such as sequence entropy, pairwise distance distributions, clustering-based diversity, or physicochemical diversity across generated samples. Including such analyses is essential, because high novelty alone can even result from generating random or biologically implausible sequences, which may not reflect true generative capability. For the comparisons in Table 2, additional methodological clarity is needed. It should be stated how many sequences each model generated, whether the sequence lengths were controlled and matched across methods, and whether the same sampling procedures were used. Without having these conditions specified, the comparisons may be difficult to interpret reliably.
Response: Thank you for your valuable comments. As the reviewer correctly pointed out, novelty measures the degree of difference between the generated peptides and known sequences, while diversity assesses the variation among the generated peptides themselves. Without evaluating diversity, it is difficult to determine whether the model is generating a variety of peptides with different structures and physicochemical properties, or merely producing small variations of a limited set of motifs.

Following your suggestion, we have dedicated the novelty and diversity analysis to Section 3.3. More specifically, we introduced diversity analysis based on both Shannon entropy and Levenshtein distance, which are two standard methods for quantifying sequence diversity. In our analysis, the entropy of each peptide sequence is calculated based on the distribution of amino acids within the sequence. The higher the entropy, the more diverse the amino acid composition of the sequence. This metric quantifies the variability in the amino acid distribution of the generated peptides. Levenshtein distance calculates the minimum number of edit operations (including insertion, deletion, and substitution) required to transform one sequence into another, effectively quantifying the similarity between two sequences. Sequences with a larger edit distance indicate significant differences in structure or amino acid composition, reflecting greater diversity. Generally, our model

achieved the highest diversity score (3.3115) according to Shannon entropy, indicating that the peptides it generates have the greatest variability in amino acid distribution. Although our model did not achieve the highest Levenshtein distance score, it derives the comparable performance with the top-2 best models (i.e., ``Latent Diffusion" and ``PPGC-DVAE"). This further emphasizes our model's ability to generate peptides with significant diversity and provides additional evidence of its robustness in generating a wide variety of AMPs.

In addition, regarding the comparison in Table 3, we have also clarified the methodological details. Specifically, the sequence alignment results between different models are consistent with those described in Section 3.2, where each model generated 512 sequences.

We sincerely appreciate the reviewer's valuable suggestion that both novelty and diversity are equally important when evaluating the generated peptides.

Comment 3: With respect to the segmentation procedure, I appreciate that additional explanation has been added, but several key details are still missing. In particular, the stride used for the sliding window has not been specified. It remains unclear whether the stride was fixed, equal to one, proportional to the window size, or randomly chosen. The stride directly affects the density of extracted fragments and the distribution of n-grams, so it must be clearly defined for the segmentation to be reproducible.
Similarly, the balancing strategy for ensuring an equal distribution of segment lengths has not been described with sufficient precision. The manuscript mentions stopping once a "target count" for each length is reached, but the basis for selecting these target counts is not explained. It is important to clarify whether these targets were derived from actual AMP length distributions, set uniformly across lengths, or chosen using another criterion. Additionally, since the window size is randomly selected between 2 and 50, there is a risk of introducing bias toward certain lengths unless this process is carefully controlled. The manuscript does not currently explain what mechanisms, if any, were used to prevent such bias or to ensure that shorter or longer fragments were not disproportionately represented.
Response: We thank the reviewer for the careful reading of our manuscript and for pointing out that the description of the segmentation procedure was not sufficiently detailed. In our actual implementation, we use a non-overlapping sliding-window scheme in which the stride is equal to the current window length. Specifically, for each protein sequence, we start from the beginning of the sequence and randomly select a window length L from the region [2,50]. We then extract the fragment from *start* to *start* + L, update *start* to *start* + L, and repeat this procedure. Thus, the stride is neither a fixed constant nor 1, but is adaptively set to the currently chosen window length, and consecutive fragments do not overlap. We have clarified the definition of the stride in the revised manuscript to ensure that the segmentation process is reproducible.

To obtain an approximately uniform length distribution of fragments, during segmentation, we randomly select a length L from the range [2, 50] as the window length. If the accumulated number of segments for the current length exceeds the target number, a new length will be randomly selected again. If the length of the remaining sequence is shorter than the selected L when the end of the original sequence is reached, the remaining part is kept as it is. The segmentation process stops once

all lengths reach their respective target counts. In other words, the target counts are uniformly assigned across all lengths from 2 to 50, rather than being determined directly by the empirical AMP length distribution. At the same time, because the random sampling of window lengths is constrained by the range [2, 50], this mechanism effectively prevents certain lengths from being oversampled and mitigates imbalance between shorter and longer fragments.

We have added a detailed description of this balancing strategy in the revised manuscript to improve the transparency and reproducibility of the segmentation process.

Response to Reviewer #4

Comment: All my concerns have been well addressed. I recommend it to be published in the journal.
Response: We sincerely thank you for your positive evaluation and recommendation for publication. We greatly appreciate the time and effort you devoted to reviewing our manuscript and for the constructive comments that helped us improve the quality of the work.

Response to Reviewer #5

Comment: Thank you for the revised manuscript. I appreciate that the authors have carefully addressed my comments.
Response: We sincerely appreciate your careful review of our revised manuscript and are grateful that you feel your comments have been adequately addressed. Your constructive feedback has been invaluable in improving the quality and clarity of the paper.
